# When High-Capacity Readers Slow Down and Low-Capacity Readers Speed Up: Working Memory and Locality Effects

**DOI:** 10.3389/fpsyg.2016.00280

**Published:** 2016-03-08

**Authors:** Bruno Nicenboim, Pavel Logačev, Carolina Gattei, Shravan Vasishth

**Affiliations:** ^1^Department of Linguistics, University of PotsdamPotsdam, Germany; ^2^Grupo de Lingüística y Neurobiología Experimental del Lenguaje, INCIHUSA, CONICETMendoza, Argentina

**Keywords:** locality, working memory capacity, individual differences, Spanish, German, ACT-R

## Abstract

We examined the effects of argument-head distance in SVO and SOV languages (Spanish and German), while taking into account readers' working memory capacity and controlling for expectation (Levy, 2008) and other factors. We predicted only *locality effects*, that is, a slowdown produced by increased dependency distance (Gibson, 2000; Lewis and Vasishth, 2005). Furthermore, we expected stronger locality effects for readers with low working memory capacity. Contrary to our predictions, low-capacity readers showed faster reading with increased distance, while high-capacity readers showed locality effects. We suggest that while the locality effects are compatible with memory-based explanations, the speedup of low-capacity readers can be explained by an increased probability of retrieval failure. We present a computational model based on ACT-R built under the previous assumptions, which is able to give a qualitative account for the present data and can be tested in future research. Our results suggest that in some cases, interpreting longer RTs as indexing increased processing difficulty and shorter RTs as facilitation may be too simplistic: The same increase in processing difficulty may lead to slowdowns in high-capacity readers and speedups in low-capacity ones. Ignoring individual level capacity differences when investigating locality effects may lead to misleading conclusions.

## 1. Introduction

When a reader or hearer is faced with a sentence containing a non-local dependency, (also called long-distance, filler-gap, or unbounded dependency) such as (1), the interpretation of the dependent (*what*) has to be delayed until the reader parses the head of the dependency (*did*). It has been argued that the delay taxes memory processes, and that processing difficulty increases with increasing distance (among others Gibson, 2000; Grodner and Gibson, 2005; Lewis and Vasishth, 2005; Vasishth and Lewis, 2006; Bartek et al., 2011; Husain et al., 2015). This increase in processing difficulty, which is reflected in longer reading times (RTs) at the head of the dependency, is known as a *locality effect* (Gibson, 2000; Lewis and Vasishth, 2005).


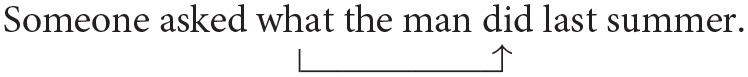


While the underlying memory processes are subject to debate, theories that predict locality effects are based on the deterioration in *some* memory processes: either an increase in integration and storage costs in Dependency Locality Theory (DLT: Gibson, 2000); or decay and interference in the case of the activation-based theory (Vasishth and Lewis, 2006). Even though there has been evidence against online language processes drawing resources from a common working memory system (Waters and Caplan, 1996; Caplan and Waters, 1999; Waters and Caplan, 2001), in recent work, Caplan and Waters (2013) argue that working memory may support retrievals in points of high processing load. Locality effects may happen in these points of high processing load, which are identified by regressive saccades and longer self-paced reading times that enable better comprehension. The interaction between individual differences in working memory capacity (WMC) and dependency resolution can shed further light on memory-based explanations of locality effects: Differential effects for different capacities can support the assumption that locality-related processing difficulty may in fact be memory based. This is not explicitly stated in DLT, but it is implied since the upper limits on storage and integration cost (Gibson and Thomas, 1999; Gibson, 2000) should depend on WMC. Furthermore, Fedorenko et al. (2006, 2013) found a reduction in performance during long-distance dependency resolution and memory dual tasks, which they interpret as the integration of non-local dependents taxing memory resources.

The relationship between WMC and retrieval processes is more explicit in the activation-based model of sentence processing (Lewis and Vasishth, 2005; Vasishth and Lewis, 2006), which is based on the Adaptive Character of Thought-Rational framework (ACT-R; see for example Anderson et al., 2004). It is assumed that a head verb triggers the retrieval from memory of its non-local dependents using cues such as number, animacy, being a wh-element, and so forth. There is no assumption of serial search in memory, but there is instead a race between the stored items (i.e., the different encoded phrases), with the most highly activated item arriving to the threshold faster and being retrieved. The latency of a retrieval thus depends on the item's level of activation. While the activation of an item decreases with a certain decay rate from the moment of its encoding, retrieval cues are used to improve the chances to identify the “right item” from memory: matching cues boost the activation of an item (while mismatching cues are penalized).

WMC can be integrated into the activation-based model by assuming that it affects the activation of items in memory differentially. One possibility is that WMC affects the decay of information from memory. This has been modeled, for example, in Just and Carpenter's (1992) CAPS, by Byrne and Bovair (1997) to explain errors after an activity that has been completed (such as forgetting the credit card in an ATM); and it has been assumed in sentence processing by, for example, Cunnings and Felser (2013) to explain the differential processing of reflexives. However, it has long been believed that it is not mainly because time passes that information in memory erodes (for a recent example, see Berman et al., 2009). Some of the findings usually associated with decay can be accommodated within *interference-based decay* (Lustig et al., 2009), which is based on the idea that the passage of time increases the likelihood that the features of an item in memory will overlap with those of a noise distribution, making them increasingly difficult to distinguish (see also Oberauer and Kliegl, 2006).

Another possibility is that WMC differentially affects spreading activation, that is, the boost of activation due to matching cues. There are at least two ways in which this could happen. One way could be because WMC modulates the total amount of activation which is shared between matching cues (see for example Cantor and Engle, 1993 for the implementation in a predecessor of ACT-R, and Daily et al., 2001; van Rij et al., 2013 for the implementation in ACT-R of number recall and pronoun resolution respectively). Another way in which WMC could affect spreading activation was suggested by Bunting et al. (2004); in their view, WMC represents susceptibility to interference. Bunting et al.'s experiment showed that individual differences are better represented if low-capacity participants activate more irrelevant cues than high-capacity participants (recall that there is a total amount of activation that is shared between the cues).

If we assume, as ACT-R does, that decay and interference both play a role (and they may be functionally related, see, e.g., Altmann and Gray, 2002), we can schematize locality effects as follows: when a dependent is parsed, it is stored in memory (together with every other phrase parsed so far in the sentence). As the distance between dependent and head increases, the representation of the dependent decays, which translates to a reduction of its level of activation. Since more recent phrases will have a higher level of activation, the correct retrieval of a non-local dependent is possible by using retrieval cues that are derived from the word eliciting the retrieval (the head), together with context and grammatical knowledge (Lewis et al., 2006). Crucially, when the amount of activation available for boosting matching cues decreases or when this activation is shared between more cues, the role of decay due to the increased distance will dominate. This would entail that the role of decay will be more pronounced for low-capacity readers.

Thus, if the source of locality effects is memory based processes (such as the ones predicted by the activation-based model or implicit in DLT), low-capacity readers should show a stronger slowdown than high-capacity ones when dependent-head distance is increased. This prediction is also supported by the following findings: When faced with difficult sentences, the disadvantage of low-WMC readers seems to increase in comparison to high-WMC ones (for garden-path vs. non-garden path sentences: Christianson et al., 2006; for comprehension reaction times in subject- vs. object-relative clauses: King and Just, 1991; Vos et al., 2001). This is also supported by evidence showing that: (a) WMC influences the probabilities of success in integrating information over a distance in a text (Daneman and Carpenter, 1980); (b) WMC is associated with the ability to maintain on-task thoughts (McVay and Kane, 2011); and (c) there is a reduction in performance during long-distance dependency resolution and memory dual-tasks (Fedorenko et al., 2006, 2013). However, this prediction is also based on the implicit assumption that RTs can be straightforwardly interpreted as indexing difficulty. We will argue that this is the case only when the retrieval of the dependent is successful. We will return to this topic and discuss the specifics of the role of WMC in the general discussion and modeling section.

Increasing dependent-head distance does not always have the same effect. Memory-driven explanations of locality effects are complicated by findings of so-called *antilocality* effects, that is evidence showing that increased distance can result in *faster* reading. For example, several studies on SOV structures (in Hindi: Vasishth, 2003; Vasishth and Lewis, 2006 and in German: Konieczny, 2000; Konieczny and Döring, 2003; Levy and Keller, 2013) showed that increasing the dependent-head distance can produce facilitation at the head of the dependency. However, such facilitation can be explained by increased expectations of the head (Levy, 2008; Levy et al., 2013; but for a memory-based explanation of facilitation see: Vasishth and Lewis, 2006; Nicenboim et al., 2015b). According to the expectation-based account, the primary source of difficulty incurred in processing a word is determined by the surprisal (negative log of the conditional probability) of a word given its context (Hale, 2001). Crucially for current purposes, this account suggests that when the distance of the dependency is increased, the appearance of the predicted head is delayed. As a consequence, the expectation of finding the head that will complete the dependency will increase monotonically. Thus, as the head is more expected, it will be processed more easily when it is encountered.

Importantly, memory- and expectation-based processes are theoretically not incompatible, and recent research (Staub, 2010; Vasishth and Drenhaus, 2011; Levy and Keller, 2013; Levy et al., 2013; Husain et al., 2014; Nicenboim et al., 2015b) shows that they may coexist. However, many of the experimental results in the literature are not easily interpretable, since increasing the distance by adding material between dependent and head systematically changes the sentences, resulting in confounding effects due to the different sentence structures engendered by the distance manipulation.

One aspect of the systematic difference between the sentences manipulated for dependency distance is the change in the linear position of the head. This is especially critical when the design argues for a speedup, since readers tend to speed up as the number of words increases (Ferreira and Henderson, 1993; Boston et al., 2008; Demberg and Keller, 2008); in (2), for example, distance is always confounded with position.

(2) a. short Someone asked **what** the man **did** last summer.
b. long Someone asked **what** the man [words that should belong somehow to the sentence] **did** last summer.

The confound between word position and distance has been addressed (see for example: Vasishth and Drenhaus, 2011; Levy and Keller, 2013) by adding the same or similar *words that should belong somehow to the sentence* before the dependency in the short version; compare now (3a) with (3b).

(3) a. short Someone [words that should belong somehow to the sentence] asked **what** the man **did** last summer.
b. long Someone asked **what** the man [words that should belong somehow to the sentence] **did** last summer.

Even though the word position confound is controlled, the new problem that arises is that the sentence structure is still consistently changed beyond the distance manipulation. If a difference is found at the head of the dependency *did* in (3), we cannot be sure whether it is a consequence of the distance manipulation or the change in the structure of the sentence. A slowdown (or a speedup) at the verb *did* in (3b) in comparison with (3a) could, in principle, have different alternative explanations. When lexical material is attached to a dependent to increase the dependent-head distance, the dependent that is retrieved in the longer version has also a richer semantic content that may produce a speedup at the verb (Hofmeister, 2007; Hofmeister and Sag, 2010; Hofmeister and Vasishth, 2014). This would be the case if *words that should belong somehow to the sentence* were, for example, a relative clause or a prepositional phrase in (3), so that the extra material is attached to *the man* in the long condition (and to *someone* in the short one). This is also exemplified in (4) from Grodner and Gibson (2005): when the distance is increased, the semantic content of the dependent also changes, namely, *the nurse from the clinic* is retrieved at the verb instead of just *the nurse*. Even though Grodner and Gibson did find locality effects, it does not rule out that the memory-driven locality effects were partially reduced by facilitation due to richer semantic content (and because of increased word position). But alternatively, the slowdown at the verb may have had independent reasons: When the dependent is more complex, it may include several nouns (*nurse* and *clinic* in the Experiment 4) that could cause encoding (Oberauer and Kliegl, 2006) and/or retrieval interference (Van Dyke and McElree, 2006), producing a slowdown at the head verb as well.

(4) Embedded verb conditions from Grodner and Gibson's (2005) experiment 2:
a. **The administrator** who the nurse **supervised** scolded the medic while …
b. **The administrator** who the nurse from the clinic **supervised** scolded the medic while …

In addition, there is evidence that preverbal material in the verbal phrase (VP) may cause a speedup at the verb, since the interposed material can help to strengthen the representation of the upcoming head by activating it through modification (as proposed by Vasishth and Lewis, 2006, and more recently Nicenboim et al., 2015b). This would be the case if *words that should belong somehow to the sentence* in (3) were an adverb such as *secretly*, so that the VP that contains the head in the long distance condition is *secretly did*, while it is only *did* in the short one (since *secretly* is attached to *asked* in the short condition). Furthermore, when the distance is increased by any manipulation, expectations may play a role (Hale, 2001; Levy, 2008): Once the reader starts parsing the embedded sentence at *what*, he or she will also start building expectations for the embedded verb; and these expectations will be different for the long and short conditions. In Levy's (2008) study, this is explained by assuming that the reader has knowledge about the grammar of the sentence, that is, he or she knows that the embedded sentence has some verb, but does not know when it will appear. The more constituents within the embedded sentence that have been integrated, the fewer possible choices there are for subsequent constituents. This means that the reader's expectation for the verb should increase as the number of integrated constituents increases. Thus, since the verb *did* in (3b) is assumed to be more expected than in (3a), it is also predicted to be processed faster.

One way to avoid many of the potential confounds and control for the differences in sentence structure is to compare each of the two experimental conditions, such as (5a) and (5b), to baseline conditions without an unbounded dependency, such as (5c) and (5d). Critically, in both short (5c) and long (5d) baseline conditions, a dependent of the verb (e.g., *something*) appears locally in the VP after the verb and remains at the same distance from the verb replacing the wh-element of the unbounded dependency conditions (5a) and (5b). In this experimental design, locality effects appear as an interaction between dependency type (unbounded vs. local, i.e., baseline), and the length of the material added immediately before the head verb (short vs. long). The sentences with local dependencies would act as baselines canceling out other effects that do not depend on the unbounded dependency. For example, if the extra material is attached to the subject of the embedded clause (*the man*), both long (unbounded and local dependency) conditions will have an argument with a richer semantic content that would require more encoding and trigger more expectations for a head verb (since the clause that starts either at the *what* or *that* is longer) than both short (unbounded and local dependency) conditions. Thus, locality effects at the critical region (*did*) would manifest as the difference between long-unbounded and short-unbounded (5b) − (5a) being larger than the difference between long-baseline and short-baseline (5d) − (5c) conditions.

(5) a. short - unbounded dependency
Someone [words that should belong somehow to the sentence] asked **what** the man **did** last summer.
b. long - unbounded dependency
Someone asked **what** the man [words that should belong somehow to the sentence] **did** last summer.
c. short - baseline (local dependency)
Someone [words that should belong somehow to the sentence] asked if the man **did** *something* last summer.
d. long - baseline (local dependency)
Someone asked if the man [words that should belong somehow to the sentence] **did** *something* last summer.

In the following experiments, we used this experimental design together with tasks that measure WMC and reading fluency in order to disentangle locality effects from potential confounds, and to find out whether locality interacts with individual differences. We used the operation span task (Turner and Engle, 1989; Conway et al., 2005) to obtain a reliable measure of WMC of our participants. We expected locality effects to be the strongest for readers with the lowest WMC readers, and we expected their magnitude to decrease with increasing WMC. One of the strengths of this type of design is that we can investigate locality effects without a priori commitments about the effect of the systematic change in the syntactic structure, that is, whether the long conditions will show a slowdown or a speedup at the critical region in comparison with the the short ones when we disregard the dependency manipulation.

It has been argued that differences in WMC may reflect differences in language experience or language skills, and not necessarily intrinsic capacity differences (MacDonald and Christiansen, 2002; Wells et al., 2009; Traxler et al., 2012), since WMC tends to correlate with many other reader characteristics.

In fact, while Traxler et al. (2005) found that WMC and syntactic complexity interacted in an eye-tracking experiment, a re-analysis of the data (Traxler et al., 2012) showed that reading speed accounted for more variation in individuals' responses than WMC. According to Traxler et al. (2012), fast readers, who read more often than slow readers, will have greater experience with language; this would in turn make them more sensitive to semantic cues in the syntactic analysis.

In order to obtain an independent measure of reading speed, we included an additional task called rapid automatized naming task (RAN: Denckla and Rudel, 1976). RAN has been shown to capture important variance associated with the processing of rapidly occurring serial information and it has been shown to predict reading speed, comprehension, and other characteristics associated with fluent reading (among others: Kuperman and Van Dyke, 2011; Araújo et al., 2015).

Norton and Wolf (2012) recently reviewed an extensive body of research that led them to consider RAN tasks “as one of the best, perhaps universal, predictors of reading fluency across all known orthographies” (p. 430). Norton and Wolf's view is that this task and reading are seen to require many of the same processes, such as eye saccade control, and the connecting of orthographic and phonological representations. By reading fluency, Norton and Wolf (2012) mean “fluent comprehension” (Wolf and Katzir-Cohen, 2001), that is, “a manner of reading in which all sublexical units, words, and connected text and all the perceptual, linguistic, and cognitive processes involved in each level are processed accurately and automatically so that sufficient time and resources can be allocated to comprehension and deeper thought” (Norton and Wolf, 2012, p. 215). Even though RAN tasks are usually used to study reading development and dyslexia, a few studies have shown that RAN is also predictive of some characteristics of reading fluency for non-college bound participants aged between 16 and 24 (Kuperman and Van Dyke, 2011), for undergrad students (Al Dahhan et al., 2014; Kuperman et al., in press), and for adults aged between 36 and 65 (van den Bos et al., 2002). In addition, some imaging studies performed in young adults have also shown that RAN and reading activate similar neural networks of neural structures (Misra et al., 2004; Cummine et al., 2015). Even though RAN has been shown to be predictive of online processes associated with word recognition, a recent study (Kuperman et al., in press) argued that RAN may not be predictive of comprehension accuracy, at least for highly proficient population such as college students. However, it may be the case that in situations of high cognitive load, more fluent readers could show an advantage in comparison with less fluent readers. The inclusion of RAN can thus help us to determine whether some participants by virtue of being fluent readers have enough resources for a more efficient use of the retrieval cues and thus overcome more easily locality effects than less fluent readers.

Since most of the evidence from locality effects and most of the evidence from antilocality effects come from SVO and SOV structures respectively, our experiments also verify whether the same account has cross-linguistic validity.

## 2. Experiment 1

### 2.1. Methods

#### 2.1.1. Participants

Seventy-nine subjects aged between 18 and 44 years old (mean 25.2 years) participated in the experiment in Argentina. All participants were native speakers of Spanish and were naïve to the purpose of the study. One additional participant was excluded from the analysis, since s/he reported that s/he suffered from a mental disorder related to memory after the experiment was conducted. Data from this experiment were collected in the same run as the self-paced reading experiment in Nicenboim et al. (2015b): the stimuli from one experiment served as filler sentences for the other experiment.

#### 2.1.2. Stimuli

The stimuli for this experiment consisted of 48 items in Spanish with four conditions following the same logic as in (5) in a two-by-two design: embedded subject length × dependency, as illustrated in (6). The embedded subject length manipulation was created by converting the proper noun of the short condition into a PP that is attached to another NP: (6a vs. 6b, and 6c vs. 6d). The dependency manipulation was created by comparing conditions with an unbounded dependency vs. local dependency (baseline) conditions, so that only the conditions with the unbounded dependencies have shorter or longer dependencies, and the baseline conditions (6c-6d) have similar structures (shorter or longer subjects) but no unbounded dependencies.

(6) a. short - unbounded dependency
a. short - unbounded dependency La hermana menor de Sofía preguntó **a quién** The younger sister of Sofia asked **who.ACC** fue que María **había saludado** en la puerta del was that María **had greeted** at the door of the colegio ayer a la tarde.
b. long - unbounded dependency
Sofía preguntó **a quién** fue que Sofia asked **who.ACC** was that la hermana menor de María **había saludado** en la the younger sister of María **had greeted** at the puerta del colegio ayer a la tarde. door of the school yesterday at the afternoon
c. short - baseline La hermana menor de Sofía preguntó si María The younger sister of Sofia asked if Maria **había saludado** a la prima de Paula en la puerta **had greeted** to the cousin of Paula at the door del colegio ayer a la tarde. of the school yesterday at the afternoon
d. long - baseline Sofía preguntó si la hermana menor de María Sofia asked if the younger sister of Maria **había saludado** a la prima de Paula en la puerta **had greeted** to the cousin of Paula at the door del colegio ayer a la tarde. of the school yesterday at the afternoon

The 48 experimental items of the current experiment were presented together with 108 experimental items for other experiments and 56 filler sentences. The sentences presented included (i) 36 items with embedded object questions and adverbs in different positions from Nicenboim et al. (2015b); (ii) 48 items with embedded object questions from an unpublished study; (iii) 24 items with object and subject experiencer psychological verbs and different word order (SVO-OVS) from an unpublished study; and (iv) 56 filler sentences with a variety of saying verbs and embedded sentences.

#### 2.1.3. Procedure

Subjects were tested individually using a PC. Participants completed three tasks at their own pace: tests to assess the individual differences in WMC (operation span task: Turner and Engle, 1989; Conway et al., 2005) and in reading fluency (rapid automatized naming: Denckla and Rudel, 1976), and a moving window self-paced reading task (Just et al., 1982).

##### 2.1.3.1. Operation span

Participants took part in an operation span task (Turner and Engle, 1989) using a software developed by von der Malsburg (2015) and used previously in von der Malsburg and Vasishth (2013). Even though variants of the reading (or listening) span task by Daneman and Carpenter (1980) have been used in many psycholinguistic studies, we chose to use the *operation* instead of the *reading* span task, since the latter is likely to measure verbal ability or reading experience as well as working memory capacity (MacDonald and Christiansen, 2002; Conway et al., 2005). We elaborate on this point below.

Even though both reading span and operation span have been defined as measures of verbal working memory (Conway et al., 2005), we think that using the operation span task presents a methodological advantage. The reading span task measures participants' abilities to do language-processing tasks, such as maintaining the phonological activation for the words in the face of competing demands from sentence processing, and thus it is not surprising that the reading span may be predictive of sentence processing phenomena (MacDonald and Christiansen, 2002). In contrast, the operation span task (described below) is further from language related tasks. And in fact, Turner and Engle (1989) motivation for the use of the operation span was that “A measure of WM should successfully transcend task dependence in its prediction of higher level cognitive functioning. That is, the memory span task could be embedded in a concurrent processing task that is unrelated to any particular skills measure and still predict success in the higher level task” (Turner and Engle, 1989, p. 129). Furthermore, a study of McVay and Kane (2011) showed some critical differences between reading and operation span task. McVay and Kane used among other measures of individual differences three complex span tasks, namely, operation, reading, and spatial span tasks. Even though the three tasks were highly correlated, the reading span task correlated with more reading comprehension tasks (and more strongly) than the operation span.

The procedure of the operation span task test was the following: At a first stage, participants had to judge the correctness of 25 simple equations. During this practice, the reaction time of Equations 10–25 was measured; the average reaction time plus two standard deviations was used as a time-out at the second stage. Having a time-out for every participant ensures that participants that are fast will not have time left to rehearse the items at the next stage of the test. At the second stage, participants had to verify equations and memorize letters (always consonants) that were shown between the equations. After each equation, a consonant was shown for 800 ms; and after a group from three to seven equation-letter successions, participants were instructed to type the letters that had appeared before in their order of presentation. During both parts of the test, participants had to read the equations and letters aloud in order to prevent vocal rehearsal strategies.

As a numeric score of individual working memory, we computed partial-credit unit scores, which indicate the mean proportion of correctly recalled items within the sets (Conway et al., 2005).

##### 2.1.3.2. Rapid automatized naming

Participants' reading fluency was operationalized using rapid automatized naming speed. Subjects that perform this task faster tend to have better reading comprehension scores, faster reading rates and their initial landing position when fixating tends to be closer to the center (among others: Howe et al., 2006; Arnell et al., 2009; Kuperman and Van Dyke, 2011; Araújo et al., 2015). Rapid automatized naming times were measured using a software developed by the first author (https://github.com/bnicenboim/py-ran-task). The procedure of the test was the following: Each subject was instructed to read a series of trials with 50 items; the items were the same set of letters or numbers that were used in Denckla and Rudel (1976): {o, a, s, d, p} and {2, 6, 9, 4, 7}. The first eight trials were composed of letters and the following eight ones of numbers. The items were displayed in five rows of ten columns and were listed in random order. Participants were instructed to start reading aloud as fast as possible immediately after pressing the spacebar, and to press it again immediately after finishing reading aloud the last item. In case they misread, they were instructed to reread only the misread item. The test started with two practice trials to familiarize the participants with the task.

##### 2.1.3.3. Self-paced reading

For the self-paced reading task all sentences were displayed in a single line and were presented in 18 pt Arial font using Linger software (http://tedlab.mit.edu/~dr/Linger/). A true-or-false comprehension task was presented after 65% of all trials in the experiment including fillers to ensure that participants had paid attention to the sentences. The statements focused on various aspects of the stimuli, and the proportion of true and false statements was balanced. For the sentences in the previous example (6) the statement was: *La hermana menor de Sofía preguntó algo*. “The younger sister of Sofía asked something,” which was true for the short conditions but false for the long ones. The statements following other experimental sentences focused on different aspects of the stimuli: the participants, the action, the setting of the action, etc. As in Nicenboim et al. (2015b), we chose to use true-or-false statements instead of yes-no questions in order to avoid long and unnatural questions.

### 2.2. Results

#### 2.2.1. Data analysis

The data analysis was conducted in the R programming environment (R Core Team, 2015), using hierarchical models (also known as mixed effects or multilevel models) in Stan (Stan Development Team, 2015b) with the R package RStan (Stan Development Team, 2015a). We fit Bayesian rather than frequentist models, which are generally fit with lme4 (Bates et al., 2014; we provide, however, the results of the frequentist models in the Supplementary Material for comparison purposes). First, hierarchical models minimize false positives when they include the maximal random effects structure justified by the design (Schielzeth and Forstmeier, 2009; Barr et al., 2013). However, such maximal frequentist models did not converge for our data and therefore had to be simplified. In contrast, their Bayesian counterpart could be fit in Stan, by using appropriate weakly informative priors for the correlation matrices (so-called LKJ priors). Third, Bayesian hierarchical models solve the multiple comparisons problem since all relevant research questions can be represented as parameters in one coherent hierarchical model (Gelman et al., 2012). This puts more burden on the hierarchical models and shifts point estimates and their corresponding intervals toward each other via “shrinkage” or “partial pooling” (see Gelman et al., 2012, for more details). Fourth, Bayesian procedures provide credible intervals rather than confidence intervals. A 95% credible interval demarcates the range within which we can be certain with probability 0.95 that the true value of a parameter lies (given the data at hand). By contrast, a frequentist confidence interval (CI) is a property of the statistical procedure and not of the parameter. The CI indicates that when the procedure is used repeatedly across a series of hypothetical data sets (i.e., the sample space), the procedure will yield intervals which contain the true parameter value in 95% of the cases (Hoekstra et al., 2014 and see Morey et al., 2016 for an extreme example of the difference between confidence and credible intervals). Thus, the frequentist CI cannot be used for inference because it tells us nothing about the uncertainty regarding the parameter's value. By contrast, the Bayesian credible interval expresses uncertainty about the parameter.

Another reason for using Bayesian models is that Bayesian procedures allow us to fit virtually any kind of distribution in a straightforward way. Residual RTs in self-paced reading are usually not normally distributed: They are limited on the left by some amount of time (i.e., the shift of the distribution), and they are highly right skewed. RTs can be reciprocal or log-transformed, but these transformations still assume that RTs are defined by their scale (mean) and shape (standard deviation), and they are unshifted (or have a shift of 0 ms). Rouder (2005) raises the concern that restricting the shift to be zero is unreasonable for response times. Unshifted distributions for *reading* times in SPR may also be unreasonable, since they do not take into account that there is a minimal amount of time that takes to read a word and press a button on the keyboard, typically around 150–250 ms. Evidence from distributional models similar to the shifted lognormal shows that shifts are nonzero and vary across participants (see, for example, Logan, 1992; Rouder et al., 2005). If distributions are shifted and analyzed as unshifted lognormal, with increasing shift, estimates of the mean artificially increase, and estimates of the standard deviation artificially decrease; and these artifacts may influence conclusions (Rouder, 2005). We decided to fit models with shifted lognormal distributions not only to avoid anti-conservative conclusions, but also to get more accurate estimates by fitting our data with a model that resembles the process that generates the data. Furthermore, when we compared the shifted lognormal distribution with unshifted distributions such as a reciprocal or a log transformation on the normal distribution, a model ranking according to the Watanabe-Akaike information criterion (or Widely applicable information criterion or WAIC; Watanabe, 2010; Vehtari and Gelman, 2014) favored the model with the shifted lognormal distribution. This may not be the only way to achieve a realistic fit to RTs; however, the shifted lognormal distribution has two key characteristics that are desirable of a RT distribution (Rouder et al., 2008): (i) it has a shift (which is absent in, for example, the ex-Gaussian distribution) and (ii) its error variance increases with mean RT (Wagenmakers and Brown, 2007). In addition, lognormal distributions are ubiquitous in nature, are well understood (Limpert et al., 2001), and are already used in psycholinguistics. We acknowledge that deeper research is needed to evaluate the advantages and disadvantages of different distributions in RTs in self-paced reading (similar to what was done for visual search by Palmer et al., 2011).

Thus we fitted a hierarchical model with a shifted lognormal distribution, allowing the shift to vary by participant. We present the posterior probability of the coefficients being positive given the data and its 95% credible interval. For all the models presented in the experiments, the predictors were sum coded (-1 and 1 for baseline and long dependency, and −1 and 1 for short and long), and covariates WMC and reading fluency were scaled and centered. In order to be able to compare the results across experiments (and regions), we report the estimates of the parameters $\hat{\delta_{k}}$ that quantify the effect size of each given coefficient k of the mixed model $\hat{\beta_{k}}$, such that $\hat{\delta_{k}}=\hat{\beta_{k}}∕\hat{\sigma }$, where $\hat{\sigma }$ is the estimated standard error of the model (as recommended by Rouder et al., 2012). Effect sizes are a dimensionless quantity (Wagenmakers et al., 2010) and depend less on the methodology (self-paced reading, eye-tracking, EEG), the language, the type of participants (students or general population), etc, than the estimates. (We provide the code of the model in the Supplementary Material.)

We checked the convergence of the models after fitting them with eight chains and 2000 iterations, half of which were the burn-in or warm-up phase. In order to assess convergence, we verified that the $\hat{R}$s were close to one, and we also visually inspected the chains (Gelman et al., 2014).

#### 2.2.2. Results of the individual differences measures

##### 2.2.2.1. Operation span

Partial-credit unit scores for the operation span test measuring WMC of the 79 participants had an average of 0.63 (*SE* = 0.01; range 0.37–0.88).

##### 2.2.2.2. Rapid automatized naming

Average character speed for the rapid automatized naming task for measuring reading fluency ranged between 1.60 and 3.45 characters∕second with an average of 2.40 (*SE* = 0.05) characters/second. The reciprocal of the averaged reading time was used as the reading fluency measure; this way a higher value represents a more skilled reader.

These two measures were not correlated for the participants of the experiment; *r* = −0.04, *CrI* (Credible Interval) = [−0.26, 0.18]. However, both were moderately correlated with the general accuracy for all the items; WMC: *r* = 0.21, *CrI* = [0.00, 0.42]; reading fluency: *r* = 0.29, *CrI* = [0.05, 0.50]. It should be noted that even though these two measures were not correlated for our subjects, who were mostly university students, it does not mean they are not correlated in the general population. The lack of correlation may be due to the so-called Berkson's paradox (Berkson, 1946), which arises when a specific part of the population is absent (in this case we can assume that people with not enough reading fluency or WMC would not attend college). However, the lack of correlation is informative in that the two measures may be tapping different underlying capacities or skills.

#### 2.2.3. Results of the self-paced reading experiment

##### 2.2.3.1. Comprehension accuracy

Participants answered correctly on average 77% (*SE* = 1) comprehension probes of the trials belonging to the experiment.

##### 2.2.3.2. Reading times

We fitted a single model for our four regions of interest using Helmert contrasts; see example (7). This type of coding ensures the interpretability of the effects of interest (length, dependency type, WMC, reading fluency, and their interaction) and allows us to detect a change in the pattern of the effects across the regions. We defined four contrasts that compare each region with the average of the preceding ones: (i) The first critical region (the auxiliary verb “había”) is first compared with the precritical region (always a proper noun), then (ii) the second critical region (a participle form of the verb), (iii) the first spillover (a preposition), and finally (iv) the second spillover (a determiner) are compared with the average of their respective preceeding regions; see Table 1. In order to account for the correlations between the regions in a single sentence, we included random effects by sentence besides by participants and items as it is usual. We included random intercepts for participants, item and sentences, and by-participants and items random slopes for length, dependency and their interaction (with their correlations).

**Table 1:** **Helmert contrasts used for both experiments**.

Precritical	−1	−1	−1	−1
Critical 1	1	−1	−1	−1
Critical 2	0	2	−1	−1
Spillover 1	0	0	3	−1
Spillover 2	0	0	0	4

**Figure 1:**
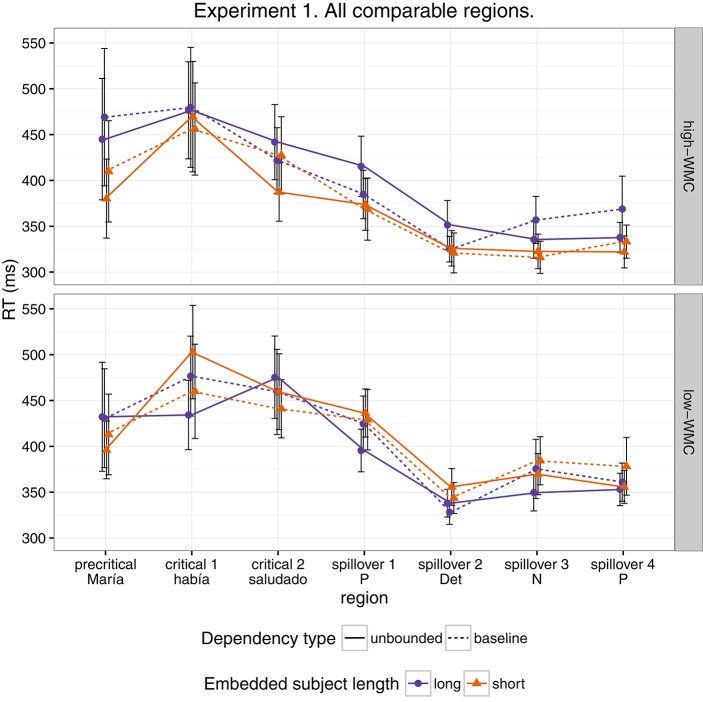
**Comparison of mean RTs for high- and low-WMC readers at each comparable region for every condition**.

**Figure 2:**
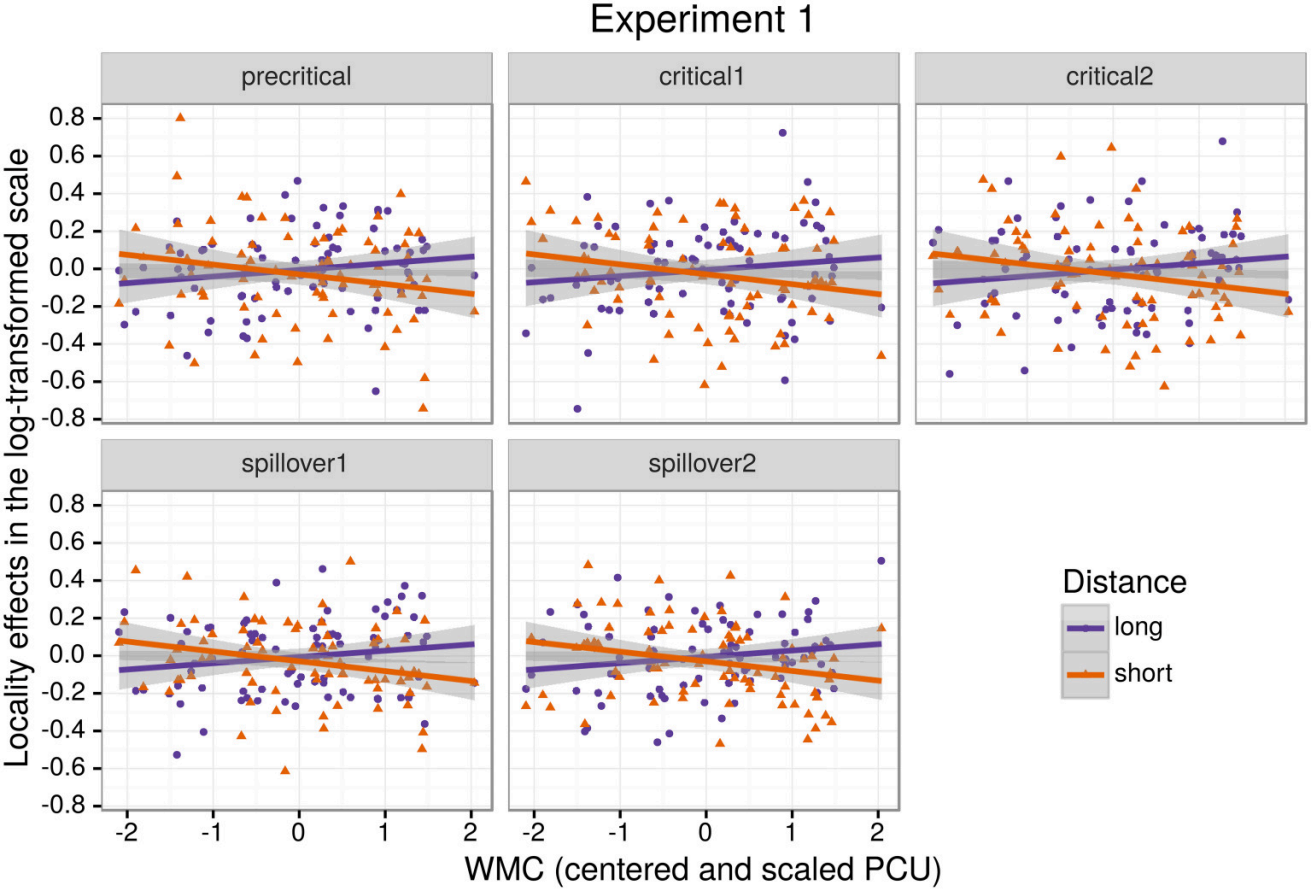
**The figure depicts the partial effect for the difference between unbounded conditions and baseline conditions, that is the (anti)locality effects, on the transformed scale of the analysis; random factors variance and effects due to reading skills were removed from the dependent variable (Hohenstein and Kliegl, 2013)**.

Figure 1 shows mean RTs for high- and low-WMC readers at each comparable region, while Figure 2 shows only the locality effects × WMC interaction.

Observations with RTs under 150 ms and above 5000 ms were removed from the data (3.84%) after checking the residuals of the model. Values below 150 ms are too fast to be reading times, and they are likely to be erroneous taps on the spacebar. If RTs that are too fast are included, the model cannot estimate the appropriate shifts in the distribution (Rouder et al., 2005).

**Figure 3:**
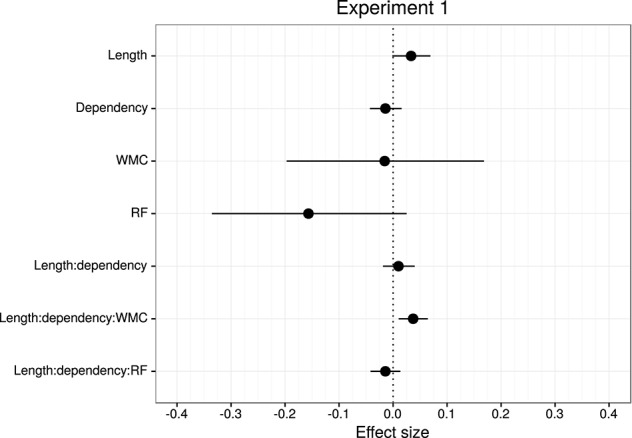
**Overview of mean and 95% credible intervals for the effect sizes of the parameters of interest for Experiment 1**.

Table 2 and Figure 3 summarize the main results of the model for the effects of reading fluency, WMC, locality (embedded subject length × dependency), and its interaction with reading fluency and WMC, including the data from all the regions of interest.

**Table 2:** **Main results for Experiment 1 (Spanish)**.

**Predictor**	**$\hat{\delta }$**	**95% CrI**		***P*($\hat{\delta }>0$)**
Length	0.03	0	0.07	0.97
Dependency	−0.01	−0.04	0.02	0.17
WMC	−0.02	−0.2	0.17	0.42
RF	−0.16	−0.34	0.03	0.04
Length:dependency	0.01	−0.02	0.04	0.74
Length:dependency:WMC	0.04	0.01	0.06	1
Length:dependency:RF	−0.01	−0.04	0.01	0.15

*WMC stands for working memory capacity and RF for reading fluency. The first column $\hat{\delta }$ shows the estimated effect size of the coefficients; the next two columns show the 2.5th and 97.5th percentiles of their posterior distribution, that is, where the effect size lies with 95% probability; and $P\left(\hat{\delta }>0\right)$ indicates the posterior probability that each coefficient is positive*.

In contrast to Null Hypothesis Significance Testing (NHST), where a sharp binary decision is made between “significant” and “non significant” effects, a Bayesian analysis allows us to compute the probability that the coefficient is positive or negative given the data. The 95% Bayesian credible interval has the interpretation that researchers often ascribe mistakenly to frequentist confidence intervals (Morey et al., 2016): it gives the range over which we can be 95% certain, given the data, that the true value of the parameter lies. This statement cannot even be made in NHST, since the true parameter is a point value with no probability distribution. A common way (Kruschke et al., 2012) to interpret the 95% credible interval is to consider an effect to be strong if 0 lies outside the interval. If 0 is included within the interval, there might still be weak evidence for an effect if the probability of the parameter being less than (or greater than) 0 may still be quite large. An example may clarify this: if the probability of the parameter being less than 0 is 0.04, i.e., $P\left(\hat{\delta }<0\right)=0.04$, this means that there is a 0.96 probability, given the data, that the parameter is negative. Here, it would be odd to say that “there is no effect” given that the posterior probability of the parameter being negative is 0.96. Accordingly, we will interpret the results as follows: if 0 lies outside the 95% credible interval, we assume that the evidence is strong that there is an effect; if 0 is included within the interval but the probability of the parameter being less than or greater than 0 ($P\left(\hat{\delta }<0\right)$ or $P\left(\hat{\delta }>0\right)$, depending on the expected sign of the effect) is high, we will say that there is weak evidence of an effect; and if the probability $P\left(\hat{\delta }<0\right)$ or $P\left(\hat{\delta }>0\right)$ is low, we will conclude that there is no evidence of an effect. For a detailed tutorial on fitting and interpreting Bayesian linear mixed models, see Sorensen et al. (2015).

The model reveals three main findings: (i) As expected, subjects with higher reading fluency scores tended to have shorter RTs (notice that even though zero is included in the credible interval, the effect size is between four and ten times larger than the rest of the effects, and 96% of its posterior probability is below zero); (ii) we did not find the hypothesized locality effects, that is, an interaction between embedded subject length and dependency type regardless of WMC; and (iii) the model shows evidence for an interaction between locality effects and WMC (embedded subject length × dependency type × WMC): For the conditions with unbounded dependencies only, the low-WMC readers showed a slight advantage for the long condition, which was reduced as WMC increased until it became an advantage for the short condition. Even though the interaction between locality effects (embedded subject length × dependency type) and reading fluency showed the predicted direction (smaller locality effects as reading fluency increases), the model shows very weak to no evidence for the effect. We do not report the interactions with the different regions in Table 1 since they show no evidence that the pattern of the effects varies across regions (including the precritical region as it can be seen in Figures 1, 2). However, nested comparisons where the models were evaluated at the different regions show that the locality × WMC interaction was mainly driven by the precritical, first critical, and spillover regions; see Table 3.

**Table 3:** **Main results for each region of Experiment 1 (Spanish)**.

**Predictor**	**$\hat{\delta }$**	**95% CrI**		***P*($\hat{\delta }>0$)**
**PRECRITICAL**				
Length	0.04	−0.01	0.08	0.94
Dependency	−0.03	−0.07	0	0.03
WMC	0.01	−0.15	0.17	0.54
RF	−0.19	−0.34	−0.02	0.01
Length:dependency	0.03	−0.01	0.07	0.93
Length:dependency:WMC	0.04	0	0.07	0.99
Length:dependency:RF	−0.04	−0.07	0	0.02
**CRITICAL1**				
Length	0.04	0	0.08	0.99
Dependency	−0.02	−0.06	0.01	0.1
WMC	0.02	−0.15	0.2	0.6
RF	−0.1	−0.26	0.06	0.1
Length:dependency	0.01	−0.03	0.04	0.66
Length:dependency:WMC	0.05	0.01	0.08	1
Length:dependency:RF	−0.02	−0.05	0.02	0.18
**CRITICAL2**				
Length	0.03	0	0.07	0.97
Dependency	−0.02	−0.06	0.01	0.1
WMC	0	−0.15	0.15	0.49
RF	−0.2	−0.35	−0.04	0.01
Length:dependency	0.02	−0.02	0.05	0.8
Length:dependency:WMC	0.02	−0.02	0.05	0.82
Length:dependency:RF	−0.01	−0.05	0.02	0.25
**SPILLOVER1**				
Length	0.02	−0.01	0.06	0.88
Dependency	0	−0.03	0.04	0.6
WMC	−0.06	−0.18	0.07	0.19
RF	−0.14	−0.27	−0.01	0.02
Length:dependency	−0.01	−0.04	0.03	0.38
Length:dependency:WMC	0.04	0.01	0.07	0.99
Length:dependency:RF	0	−0.03	0.03	0.5
**SPILLOVER2**				
Length	0.01	−0.03	0.05	0.76
Dependency	0.01	−0.03	0.05	0.75
WMC	−0.04	−0.16	0.08	0.24
RF	−0.18	−0.3	−0.06	0
Length:dependency	−0.01	−0.04	0.03	0.36
Length:dependency:WMC	0.01	−0.02	0.05	0.72
Length:dependency:RF	0	−0.03	0.04	0.59

*WMC stands for working memory capacity and RF for reading fluency. The first column $\hat{\delta }$ shows the estimated effect size of the coefficients; the next two columns show the 2.5th and 97.5th percentiles of their posterior distribution, that is, where the effect size lies with 95% probability; and P($\hat{\delta }$ > 0) indicates the posterior probability that each coefficient is positive*.

It is also worth noting that the length of the embedded subject had an effect on the RTs at the regions of interest, irrespective of the dependency manipulation. This effect would have been confounded with locality in the absence of appropriate baselines. This raises the concern that some of the previous studies that reported a main effect of locality could in principle have been reporting the effect of increasing the complexity of the subject that appeared prior to the verb.

### 2.3. Discussion

For this experiment, even though we found an effect of embedded subject length, we did not find evidence of locality effects (an embedded subject length × dependency type interaction) across the board. Furthermore, even though an interaction between WMC and locality effects was expected, the interaction was predicted in the opposite direction. We predicted that low-capacity participants would show the strongest locality effects, while counter-intuitively, in our experiment it was the high-WMC participants that showed the strongest locality effects (the largest difference between *(long unbounded* − *long baseline)* and *(short unbounded* − *short baseline)*), while low-WMC showed antilocality effects; as shown in Figures 1, 2.

This interaction seems counterintuitive because theories that predict locality effects would not predict that high-WMC participants would show stronger locality effects. Locality effects are hypothesized to be a behavioral response to either the use of more computational resources (Gibson, 2000), or higher retrieval costs due to more interference and decay (Lewis and Vasishth, 2005) when the distance between head and argument is increased. However, the speedup of low-WMC readers can be accounted for by adding two intuitively plausible assumptions to memory-based explanations, namely, that low-capacity readers experience retrieval failures more frequently than high-capacity readers, thus leading to unresolved dependencies and an incomplete sentence representation compatible with good enough processing (Ferreira and Patson, 2007); and that retrieval failures are faster on average than complete retrievals. We provide further evidence supporting this claim in the next experiment and the modeling section.

Furthermore, reading fluency correlated with comprehension accuracy (as strongly as WMC) for this experiment, and participants with higher scores in reading fluency tended to read faster the regions of interest. However, we found very weak to no evidence favoring the hypothesis that fluent readers would overcome more easily locality effects than less fluent readers.

While the pattern showing stronger locality effects for high-WMC participants begins at the precritical region (a proper noun that is either the subject or the last part of it) before the verb, memory driven locality effects are predicted to appear no sooner than the verb. However, pre-verbal locality effects have been detected also in Vasishth and Drenhaus's (2011) study, and they also appeared in some degree in the next experiment. This phenomenon will be addressed in the general discussion.

## 3. Experiment 2

The second experiment attempts to replicate Experiment 1 using SOV structures in German, in contrast to the SVO structures in Spanish of the previous experiment. The main objective of the second experiment was to verify whether the same account for the findings of Experiment 1 is valid for an SOV language. This is important because SVO structures seem to trigger mostly locality effects at the head verb (among others Grodner and Gibson, 2005; Lewis and Vasishth, 2005; Vasishth and Lewis, 2006; Demberg and Keller, 2008; Bartek et al., 2011), while SOV structures seem to trigger either antilocality effects (Konieczny, 2000; Konieczny and Döring, 2003; Vasishth, 2003; Vasishth and Lewis, 2006; but see Safavi et al., Submitted) or both locality and antilocality (Vasishth and Drenhaus, 2011; Levy and Keller, 2013; Husain et al., 2014). It was therefore important to verify whether the same results can be obtained with the same manipulation irrespective of the OV/VO order.

### 3.1. Methods

#### 3.1.1. Participants

Seventy-two subjects aged between 17 and 43 years old (mean 24.6 years) were recruited using ORSEE (Greiner, 2004) at the University of Potsdam, Germany. All participants reported to be native speakers of German and were naïve to the purpose of the study. Three other participants had to be removed from the data: one subject answered randomly at the operation span task, another subject answered the comprehension questions at chance level, and the data of a third participant was lost due to technical reasons.

#### 3.1.2. Stimuli

Similarly to Experiment 1, the stimuli for this experiment consisted of 48 items in German with four conditions in a two-by-two design: embedded subject length × dependency (see Example 8).

For this experiment, the embedded subject length manipulation was created by changing the determiner (*die*) of the noun phrase of the short condition with a longer genitive phrase such as *Marias äußerst kaltschnäuzige*, “Mary's extremely uncaring”: (8a vs. 8b, and 8c vs. 8d). The dependency manipulation was created as in Experiment 1 by comparing conditions with an unbounded dependency vs. local dependency (baseline) conditions. Thus, conditions (8a–8b) were compared with two baseline conditions (8c–8d) with similar structure, but that lacked the unbounded dependency: The dependent of the verb *jemanden* (someone.ACC) appeared at the same distance of the verb in both short and long baseline conditions.

(8) a. short - unbounded dependency
Marias äußerst kaltschnäuzige Lehrerin fragte,
Mary's extremely uncaring teacher asked
**wen** die Mutter gestern beim Treffen
**who.ACC** the mother yesterday at.the meeting
**angeschrien hat** mit schriller Stimme.
**yelled had** with shrill voice
b. long - unbounded dependency
Die Lehrerin fragte, **wen**
The teacher asked **who.ACC**
Marias äußerst kaltschnäuzige Mutter gestern
Mary's extremely uncaring mother yesterday
beim Treffen **angeschrien hat** mit schriller
at.the meeting **yelled had** with shrill
Stimme.
voice
c. short - baseline
Marias äußerst kaltschnäuzige Lehrerin fragte,
Mary's extremely uncaring asked teacher
ob die Mutter jemanden beim Treffen
if the mother someone.ACC at.the meeting
**angeschrien hat** mit schriller Stimme.
**yelled had** with shrill voice
d. long - baseline
Die Lehrerin fragte, ob
The teacher asked if
Marias äußerst kaltschnäuzige Mutter jemanden
Mary's extremely uncaring mother someone
beim Treffen **angeschrien hat** mit schriller
at.the meeting **yelled had** with shrill
Stimme.
voice

The 48 experimental items of the current experiment were presented together with 98 experimental items belonging to experiments from unpublished studies. The sentences presented included (i) 32 items with subject and object relative clauses attached to the subject or the object of sentences; (ii) 42 items with attachment ambiguity involving dative and genitive noun phrases; and (iii) 24 items that contrasted personal and demonstrative pronouns.

#### 3.1.3. Procedure

The procedure was the same as the one used in Experiment 1, with the exception that comprehension questions appeared after every trial in the self-paced reading experiment.

### 3.2. Results

#### 3.2.1. Results of the individual differences measures

##### 3.2.1.1. Operation span

Partial-credit unit scores for the operation span test measuring WMC of the 72 participants had an average of 0.63 (*SE* = 0.02; range 0.28–0.92).

##### 3.2.1.2. Rapid automatized naming

Average character speed for the rapid automatized naming task for measuring reading fluency ranged between 1.43 and 3.61 characters∕second with an average of 2.64 (*SE* = 0.06) characters/second. As in Experiment 1, the reciprocal of the averaged reading time was used as the reading fluency measure.

As in Experiment 1, these two measures were not correlated for the participants of the experiment; *r* = 0.02, *CrI* = [−0.23, 0.27]. In contrast with the previous experiment, only WMC was correlated with the general accuracy for all the items; WMC: *r* = 0.42, *CrI* = [0.23, 0.60]; reading fluency: *r* = 0.01, *CrI* = [−0.24, 0.27].

#### 3.2.2. Results of the self-paced reading experiment

##### 3.2.2.1. Comprehension accuracy

Participants answered correctly on average 80% (*SE* = 1) comprehension probes of the trials belonging to the experiment.

##### 3.2.2.2. Reading times

**Figure 4:**
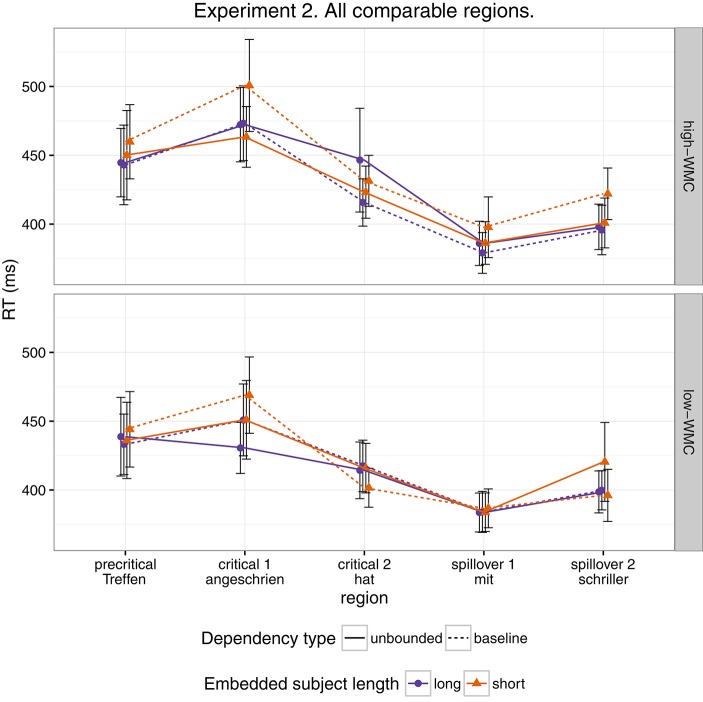
**Comparison of mean RTs for high- and low-WMC readers at each comparable region for every condition**.

**Figure 5:**
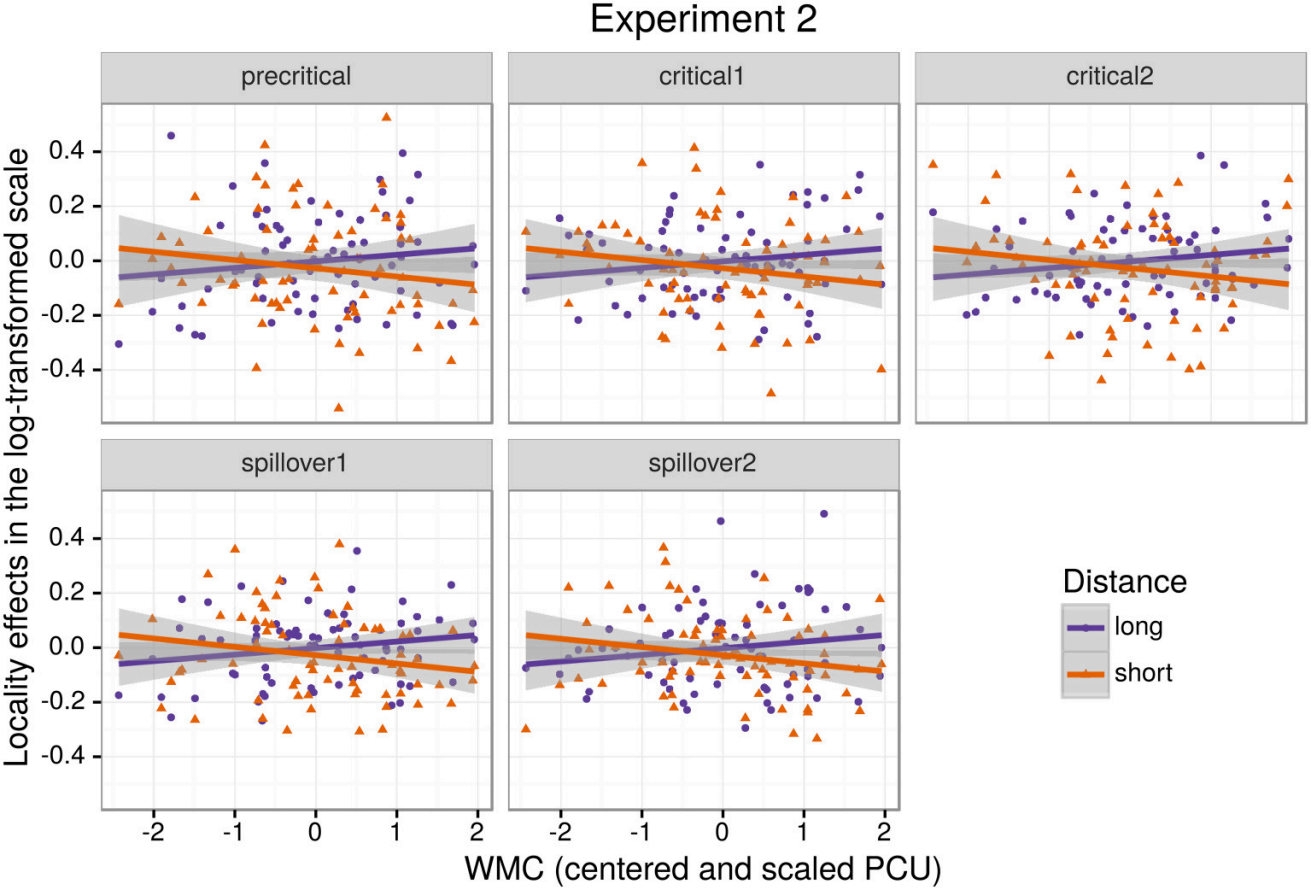
**The figure depicts the partial effect for the difference between unbounded conditions and baseline conditions, that is the (anti)locality effects, on the transformed scale of the analysis; random factors variance and effects due to reading skills were removed from the dependent variable (Hohenstein and Kliegl, 2013)**.

As for Experiment 1, we fitted a single model for our four regions of interest (9) using Helmert contrasts. Figure 4 shows mean RTs for high- and low-WMC readers at each comparable region, while Figure 5 shows only the locality effects × WMC interaction.

(9) …fragte {wen; ob} {die; Marias äußerst
…asked {who.ACC; if} {the; Maria's extremely
kaltschnäuzige} Mutter gestern beim | Treffen |
uncaring} mother yesterday at.the | meeting |
| precritical |
angeschrien | hat | mit | schriller | …
shouted | have | with | shrill | …
critical 1 | critical 2 | spillover 1 | spillover 2 |

As in Experiment 1, RTs under 150 ms and above 5000 ms were removed from the data (2.83% of the observations).

**Figure 6:**
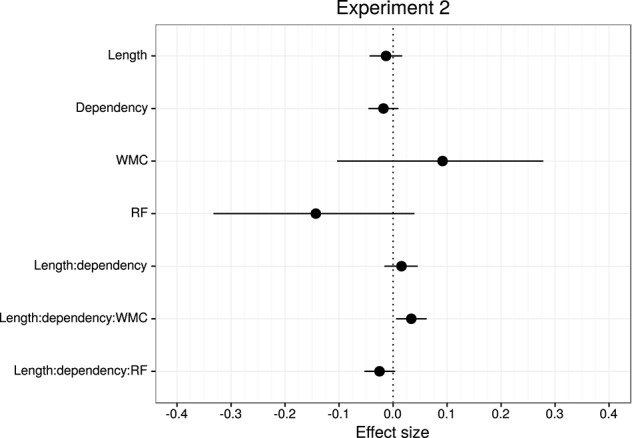
**Overview of mean and 95% credible intervals for the effect sizes of the parameters of interest for Experiment 2**.

Table 4 and Figure 6 summarize the main results of the model for the effect of reading fluency, WMC, locality effect (embedded subject length × dependency), and its interaction with reading fluency and WMC, including the data from all the regions of interest. We omitted the interactions with the different regions since the effects of interest had the same pattern in all the regions. Table 5 summarizes the results from nested comparisons where the models were evaluated at the different regions.

**Table 4:** **Main results for Experiment 2 (German)**.

**Predictor**	**$\hat{\delta }$**	**95% CrI**		***P*($\hat{\delta }>0$)**
Length	−0.01	−0.04	0.02	0.2
Dependency	−0.02	−0.05	0.01	0.11
WMC	0.09	−0.1	0.28	0.83
RF	−0.14	−0.33	0.04	0.06
Length:dependency	0.02	−0.02	0.05	0.85
Length:dependency:WMC	0.03	0.01	0.06	0.99
Length:dependency:RF	−0.03	−0.05	0	0.04

*The first column $\hat{\delta }$ shows the estimated effect size of the coefficients; the next two columns show the 2.5th and 97.5th percentiles of their posterior distribution, that is, where the effect size lies with 95% probability; and $P\left(\hat{\delta }>0\right)$ indicates the posterior probability that each coefficient is positive*.

**Table 5:** **Main results for each region of Experiment 2 (German)**.

**Predictor**	**$\hat{\delta }$**	**95% CrI**		***P*($\hat{\delta }>0$)**
**PRECRITICAL**				
Length	0.01	−0.03	0.05	0.63
Dependency	−0.03	−0.07	0.01	0.07
WMC	0.08	−0.1	0.26	0.82
RF	−0.14	−0.32	0.05	0.07
Length:dependency	0.04	0	0.07	0.97
Length:dependency:WMC	0.03	−0.01	0.07	0.95
Length:dependency:RF	−0.02	−0.06	0.02	0.14
**CRITICAL1**				
Length	−0.02	−0.06	0.02	0.14
Dependency	−0.06	−0.1	−0.03	0
WMC	0.14	−0.02	0.3	0.96
RF	−0.12	−0.29	0.03	0.06
Length:dependency	0.01	−0.03	0.05	0.74
Length:dependency:WMC	0.03	−0.01	0.06	0.92
Length:dependency:RF	−0.05	−0.08	−0.01	0
**CRITICAL2**				
Length	0	−0.04	0.03	0.42
Dependency	−0.01	−0.05	0.03	0.37
WMC	0.11	−0.04	0.26	0.93
RF	−0.11	−0.26	0.04	0.07
Length:dependency	0	−0.04	0.04	0.47
Length:dependency:WMC	0.05	0.01	0.08	0.99
Length:dependency:RF	−0.01	−0.04	0.03	0.37
**SPILLOVER1**				
Length	−0.01	−0.05	0.03	0.3
Dependency	0.02	−0.03	0.06	0.78
WMC	0.1	−0.06	0.27	0.9
RF	−0.17	−0.33	0	0.02
Length:dependency	0.01	−0.03	0.05	0.75
Length:dependency:WMC	0.01	−0.02	0.05	0.75
Length:dependency:RF	−0.02	−0.05	0.02	0.19
**SPILLOVER2**				
Length	−0.04	−0.07	0	0.02
Dependency	0	−0.04	0.03	0.42
WMC	0.07	−0.09	0.24	0.81
RF	−0.18	−0.35	−0.02	0.02
Length:dependency	0.01	−0.03	0.05	0.7
Length:dependency:WMC	0.04	0	0.07	0.98
Length:dependency:RF	−0.02	−0.05	0.02	0.15

*WMC stands for working memory capacity and RF for reading fluency. The first column $\hat{\delta }$ shows the estimated effect size of the coefficients; the next two columns show the 2.5th and 97.5th percentiles of their posterior distribution, that is, where the effect size lies with 95% probability; and $P\left(\hat{\delta }>0\right)$ indicates the posterior probability that each coefficient is positive*.

The models reveal the following: As in Experiment 1, even though it is with less certainty, subjects with higher reading fluency scores tended to have shorter RTs.

In addition, and as in the previous experiment, we did not find the hypothesized locality effects in this experiment. The models, however, show evidence for an interaction between locality effects and WMC. This interaction has the same pattern in all regions of interest. The resulting effect is similar to the one of Experiment 1, even though the underlying pattern is different (see Figure 4): the effect was mainly driven by a speedup in long baseline conditions in comparison with short baseline conditions. This speedup was reduced as WMC decreased until it became an advantage for the short condition for low-WMC readers; compare the figures depicting the effects for high- and low-WMC in Experiment 2 (Figure 5) with Experiment 1 (Figure 2).

We also found some evidence for a three-way interaction between embedded subject length, dependency type, and reading fluency, with the same direction as in Experiment 1, that is, decreasing locality effects as the score of reading fluency increases. The interaction had the following pattern: For the unbounded dependency conditions, as reading fluency increased, RTs at the long condition decreased in comparison with the RTs at the short condition; while for the baseline conditions this pattern was reversed.

#### 3.3. Discussion

We found a dependency type × embedded subject length × WMC interaction, which had the same sign as in the previous experiment. However, while in Experiment 1 the effect seemed to be caused by the difference between the unbounded dependency conditions, in Experiment 2, the effect was mainly caused by a difference between the baseline conditions. In contrast to the Spanish stimuli, the subject did not immediately precede the verb in the German stimuli and therefore had to be retrieved from memory. Since the long conditions appear together with a more informative and salient subject, and the encoding of the longer subjects seems to have not spilled over the head verb; it may be the case that the subject retrieval is faster (Hofmeister, 2007; Hofmeister and Vasishth, 2014), thus leading to a speedup in both long conditions (both unbounded dependency and baseline conditions).

But crucially, the dependency type × embedded subject length × WMC interaction had the same direction and similar magnitude as in Experiment 1, that is, high-WMC participants showed the largest difference between *long unbounded* − *long baseline* and *short unbounded* − *short baseline*, while this difference is inverted for low-WMC readers. This outcome allows us to give the same interpretation to the results of the current experiment: high-WMC readers showed locality effects and low-WMC readers showed a speedup, which we argue that it is associated with a higher proportion of failure in retrieval in the long unbounded dependency condition.

In contrast with Experiment 1, reading fluency did not show a correlation with comprehension accuracy (while only WMC did). Similarly to the first experiment, however, participants with higher scores in reading fluency tended to read the critical region faster. In addition, we found somewhat stronger evidence favoring the hypothesis that fluent readers would overcome locality effects more easily than less fluent readers.

## 4. General discussion

We found no evidence for locality effects across the board in either experiment, that is, no evidence for an interaction between dependency type and embedded subject length independent of individual differences in WMC. However, we did find evidence for an interaction between locality effects and WMC (dependency × embedded subject length × WMC) for both Spanish and German experiments. Even though there were differences in how the three-way interaction was produced between the two experiments, this may be due to the differences in the overall structure of the sentences, namely, SVO and SOV structures (and see the previous discussion). More importantly, when the differences are controlled via baselines, we see an interaction with the same (counterintuitive) pattern in both experiments: high-WMC readers showed the strongest locality effects that were reduced with decreasing WMC and eventually changed direction, such that low-capacity readers showed a speedup effect.

The speedup of low-capacity readers is in line with independent evidence showing that in some cases high working memory load may lead to faster RTs: Van Dyke and McElree (2006) found that readers showed shorter RTs (together with lower comprehension accuracy) when a memory load was present in comparison with the conditions without the memory load. Furthermore, our findings are also compatible with studies showing that low-WMC subjects may take less time when ambiguities are present (at the expense of their accuracy) than high-WMCs (MacDonald et al., 1992; Pearlmutter and MacDonald, 1995).

It should be underscored that, unlike Just and Carpenter (1992), we do not argue that the effect of WMC is directly on mechanisms specific to language, such as parsing rules. We argue instead that the effect of WMC is on the retrieval of the dependents, which we assume is driven by the same cognitive mechanisms as retrieval outside sentence processing. There is a great deal of evidence suggesting that high-WMC participants tend to do better on tasks that involve retrieval in comparison with low-WMC ones, particularly under conditions of interference: for example, Conway and Engle (1994) found that high- and low-WMC individuals differed in retrieval efficiency only when items were associated with multiple cues (which caused more interference). In a study by Kane and Engle (2000), participants were shown a list of category exemplars followed by a distractor activity. After the distractor task, the participants were instructed to recall the category exemplars. Kane and Engle found that all participants recalled a similar number of words on the first trial but that low-WMC individuals recalled fewer items than high-WMC individuals as the task progressed. Kane and Engle concluded that low-capacity individuals were more susceptible to the buildup of proactive interference than were high-capacity ones. Conway et al. (2001) extended the investigation of the cocktail party phenomenon, the situation in which one can attend to only part of a noisy environment, but stimuli such as one's own name can suddenly capture attention. While previous investigations have shown that approximately 33% of the participants hear their name in an unattended, irrelevant message channel, Conway et al. found that 65% of low-WMC participants did detect their name in contrast with 20% of high-WMC ones. This result also suggests that low-WMC are also more susceptible to interference. Kane et al. (2001) reported similar differences in an antisaccade paradigm, which presents a conflict between task goals and visual cues. High-WMC participants made fewer errors, they recovered from these errors more rapidly, they initiated antisacades more quickly, and they identified targets more quickly than did low-WMC participants.

Besides ACT-R, two recent theories of WMC posit a role of individual differences in differential effects at retrieval: Unsworth et al. (Unsworth and Engle, 2007; Unsworth et al., 2009) have recently suggested a dual-component framework for interpreting individual differences in WMC. In this framework, WMC partially reflects differences in attention control abilities together with retrieval abilities in which information that could not be maintained in the focus of attention (due to distraction and/or capacity constraints) is retrieved via a cue-dependent search process. In addition, Oberauer et al. (2012) have postulated a computational model, “serial order in a box - complex span” or SOB-CS (an extension of C-SOB; Farrell, 2006; Lewandowsky and Farrell, 2008, which originated as SOB; Farrell and Lewandowsky, 2002), where capacity is limited only by interference between representations. One of the individual differences that the model assumes is a parameter that determines the degree of discriminability between retrieval candidates.

In our view, non-local dependency resolution is a case where the individual differences in WMC may play a role: an argument that is no longer in the focus of attention has to be retrieved from memory, using information from the verb that is retrieved online, and after the parser has encoded a variable amount of lexical material that can produce interference together with either pure time-based decay or interference-based decay.

However, we must acknowledge that recent findings raise the concern that WMC may have limited value for explaining individual differences in linguistic contexts. A recent study by Van Dyke et al. (2014) replicated Van Dyke and McElree (2006) while including a battery of tests for measuring individual differences as well. This recent study showed that while high-span participants read more slowly in the conditions with high cognitive load and showed higher accuracy in comparison with low-span participants, the effect of WMC may be spurious. When receptive vocabulary was included in the analysis, it showed the same effects previously attributed to WMC, revealing that the participants with better scores in the vocabulary task were more affected by the interference during online reading. Similarly, a study of Traxler and Tooley (2007) investigating syntactic ambiguity showed that vocabulary size predicted the degree to which readers were disrupted by the syntactic misanalysis for several eye-tracking measures; while WMC was only a marginal predictor for total reading times. In addition, Long et al. (2008) study of recollection and familiarity of previously read sentences showed that only individual differences of readers' background knowledge was predictive of better performance but not WMC (but neither neither print exposure or vocabulary size). Long et al. (2008) argued that because retrieval cues were minimal, access to the text representation depended more on the reader's background knowledge than on the reading skills or WMC of the participants.

Our results do not rule out the possibility that retrieval processes in sentence processing are based on different mechanisms which are independent of WMC, and that the effect that we found is due to WMC being a proxy for other individual differences such as robustness of lexical representations (Traxler and Tooley, 2007; Van Dyke et al., 2014). This is a valid criticism, but it affects any experiment that includes individual differences. No matter how extensive the battery of tasks, there is always the possibility that a predictor is in fact a proxy for another unmeasured predictor.

In addition, the locality effects × WMC interaction in the two experiments should not be dismissed as a simple speed-accuracy trade-off. It is a well known phenomenon that accuracy deteriorates with increasing speed (see for example, Pachella, 1974, and more recently, Heitz, 2014). This general phenomenon, however, does not explain why low-WMC participants would decide to sacrifice accuracy for speed even to a rate that is higher than when there is a lower cognitive load (i.e., a shorter dependency). Furthermore, it also does not explain what mechanisms low-WMC participants may have used to identify the high-cognitive load conditions in order to speed up.

We suggest that the locality effects of the high-WMC readers and the speedup of the low-WMC readers can be explained by adding two assumptions to memory-based explanations, namely, (i) that failures of the retrieval of the dependent (the *wh*-element in this case) are more frequent in low-WMC participants than in high-WMC ones; and (ii) that retrieval failures are faster on average than complete retrievals.

The locality effects × WMC interaction in the two experiments may be related to some type of good-enough parsing strategy (Ferreira et al., 2002; Ferreira and Patson, 2007), where low-WMC readers failed to achieve a complete and fully specified representation of the sentence more often when faced with the long unbounded dependency condition. Without the possibility of re-reading, and since the comprehension questions were not targeting exclusively whether the dependency was understood, low-WMC readers may have failed in many cases to retrieve the dependent and continued reading.

In other words, we speculate that the average time *T* for the completion of a dependency is determined by:

$$\begin{array}{c} T=T_{baseline}+P_{retrieval}\cdot T_{retrieval}+\left(1-P_{retrieval}\right)\cdot T_{failure} \end{array}$$

while the proportion of completed retrievals *P*_*retrieval*_ is higher for high-WMC readers in comparison with low-WMC readers when the dependent-head distance is increased; and *T*_*retrieval*_ at a long dependency is larger than *T*_*retrieval*_ at a short dependency.

Notice, however, that without the proportion of completed retrievals (*P*_*retrieval*_) for each case, the model previously presented is unidentifiable. The proportion of completed retrievals should be linked to the accuracy of the comprehension of the dependencies.

There is some evidence that high-WMC outperformed low-WMC in general comprehension in this experiment; but we could not target the comprehension of the dependencies in the experimental stimuli. The true-or-false statements used in both Experiment 1 and 2 (as in Nicenboim et al., 2015b) included many aspects of the stimuli to verify that participants paid attention to the sentences, but they did not target exclusively whether the dependency was understood. Since the dependencies included a wh-argument, comprehension questions would ideally need to verify unnatural constructions, namely, whether it is true that, for example, “Maria greeted whom.” However, preliminary data from our lab (Nicenboim et al., 2015a), where the stimuli allowed for more informative question-response accuracy, suggest that at least for interference effects in relative clauses, both low-WMC and high-interference conditions seem to provoke more retrieval failures.

In the following section we present simulations based on the ACT-R framework to illustrate in which situations and under which assumptions our hypothesis holds.

Regarding the effect of reading fluency on locality effects, the experiments presented some weak evidence favoring the hypothesis that fluent readers may overcome locality effects more easily than less fluent ones. The evidence is rather weak for the following reasons: Reading fluency predicted comprehension accuracy in Experiment 1, where it interacted very weakly with locality effects and with much uncertainty. In contrast, reading fluency did not predict comprehension accuracy in Experiment 2, while it interacted more strongly with locality effects and with less uncertainty. Given the similarity between the experiments, it is hard to explain the discrepancies.

To some degree in Experiment 1 and with more uncertainty in Experiment 2, the pattern showing stronger locality effects for high-WMC participants begins at the precritical region before the verb. Memory driven locality effects, however, are predicted to be triggered by a retrieval process that would start presumably no sooner than the verb. One possible explanation proposed by Vasishth and Drenhaus (2011) is that the verb phrase may have already been built when the proper noun preceding the verb is processed. This assumption is consistent with Levy's (2008) expectation-based account, because the parser can deduce that the verb will appear immediately afterwards and thus anticipate the retrieval process.

It should be noted that expectations were controlled only under the simplifying assumption that given that a clause has a finite length, the probability that the next word will be the subcategorizing verb rises as the number of words after finding the wh-element increases. In a way, this is similar to the increasing hazard function proposed for visual search by Peterson et al. (2001). A more formal verification could not be conducted, since the sentences used for our two experiments were too complex for a correct parsing of a probabilistic top-down parsing (Roark, 2001; Roark et al., 2009) trained with Spanish (Moreno et al., 2003) and German treebanks (Brants et al., 2004). Even after unlexicalizing the treebanks, the parser failed to identify the structure of the sentences used in our stimuli. However, given that the speedup occurred for low-span readers in sentences with long dependencies, assuming that increasing the length of the dependencies still caused an increase in expectations beyond the control of the baseline would require the implausible assumption that low-span readers are better at making predictions than high-span readers.

## 5. Modeling

Even though both the activation-based account and DLT would intuitively predict that increasing the distance between dependent and head should have produced a slowdown (once expectations are controlled), our results do not show a main effect of locality and only an interaction with WMC. Thus, we first verified that the activation-based account in fact predicts locality effects and stronger effects for low-span readers using the ACT-R framework (see for example Anderson et al., 2004). ACT-R is a general cognitive architecture used to model a vast variety of cognitive phenomena; for our purposes, however, the relevant aspect of the architecture is that it can model the retrieval of items stored in memory. In order to simplify our models, we used only the equations that determine the probability and latency of a retrieval and not the full framework. In this section, we tested different implementations of WMC with the “default” ACT-R equations and we show that, no matter what the parameter settings are, they fail to account qualitatively for the results. Therefore, we tentatively suggest that a basic assumption about the relationship between latencies and activation needs some reconsideration; we propose that items in memory with an activation below a certain threshold may show shorter latencies because of an early aborting of the retrieval process.

The exact predictions of the ACT-R implementation of the activation-based account will depend on the exact syntactic structure and the type of parser that is assumed together with the values of the ACT-R parameters. In addition, it cannot at present accommodate certain aspects that seem to have an uncontroversial effect in language, such as expectations (Hale, 2001; Levy, 2008). Thus, we focused on the explanation of (anti-)locality effects, (i.e., the interaction distance × dependency), which was the theoretical comparison of interest; and we did not investigate the underlying processes that generated the reading times for each condition (see Introduction).

In this framework, the latency of the retrieval of an item from memory is assumed to be a function of the item's activation value *A*:

(1) $$\begin{array}{c} Latency=F\cdot e^{-A} \end{array}$$

where F (the latency factor) is a scaling constant.

After verifying that ACT-R did not predict that other noun phrases would be mistakenly retrieved, we focused only on the retrieval of the wh-element. At the moment of retrieval, the activation *A* is calculated as the sum of (i) a *base level activation BA* that depends on the previous use of the item (i.e., the number of previous retrievals and the time passed since those retrievals); (ii) *spreading activation S* that depends on a limited amount of source activation *W* that is shared between all other items with features that match the retrieval cues; (iii) a penalty component for mismatching features (that we omit from the following equation); and (iv) a random noise component ϵ (that follows a logistic distribution with a mean of zero and scale σ):

(2) $$\begin{array}{c} A=BA+S+\epsilon \end{array}$$

Locality effects affect only the base level activation due to decay; in our specific case, the base level activation of the wh-element can be described as:

(3) $$\begin{array}{c} BA=log\left(t^{-d}\right)+\beta \end{array}$$

where *d* is the decay rate, *t* is the time since the encoding of the wh-element, and β is the base-level constant.

The equation for the spreading activation S ensures that the wh-element would be retrieved due to the boost of activation produced by the unique matching features. For simplicity, we can assume that the wh-element has a unique feature that distinguishes it from the other four competitor NPs (in example 6: Sofía, the younger sister, the younger sister of Sofía, and María), namely being +*wh*, and two non-unique features (+*animate* and +*NP*) that it does share with the other NPs. The spreading activation of the wh-element is a function of the source activation *W*, and the weighted sum of the strength of association of the cues. The source activation is usually set to one, but it can also vary by participants (Daily et al., 2001), and it is divided between the cues. In the present case, this can be simplified as:

(4) $$\begin{array}{l} S=W\;\cdot \;[w_{wh}\;\cdot \;(MAS-\log(1))+w_{anim}\;\cdot \;(MAS-\log(5))\\ \;+w_{NP}\cdot (MAS-\log(5))] \end{array}$$

where *MAS* is the maximum associative strength; and *w*_*wh*_, *w*_*anim*_, and *w*_*NP*_ are the weights given to the cues +*wh*, +*animate*, and +*NP*, and must sum to one. The maximum associative strength is subtracted by the natural logarithm of the number of competing items in memory that match a given cue plus one. *MAS* is an arbitrary value, which is usually fixed since it trades off with *F* (Schneider and Anderson, 2012). We fixed this parameter to two since the difference between *MAS* and *log*(*matchingcues*+1) must be always positive in ACT-R. The three summands of the previous equation represent three features that match with three retrieval cues: The first summand represents the unique feature +*wh*, which ensures the highest value of *S* for the wh-element, and the next two summands represent the features +*animate* and +*NP*, which are shared with four competitors (hence log(5), as there are five noun phrases in total). (The spreading activation equations of the competitor noun phrases would have only the last two summands; and their activation would be reduced further by a penalty component that is also subtracted from their total activation).

WMC has been assumed to either affect the decay rate or affect in some way the spreading activation, that is, the activation shared between the retrieval cues (see the Introduction section). We simulated these possibilities by using standard ACT-R parameters from sentence processing (Lewis and Vasishth, 2005; Vasishth and Lewis, 2006), except for *MAS*, the latency factor, and the base levels constant that were adjusted to achieve realistic latencies based on previous studies.

**Figure 7:**
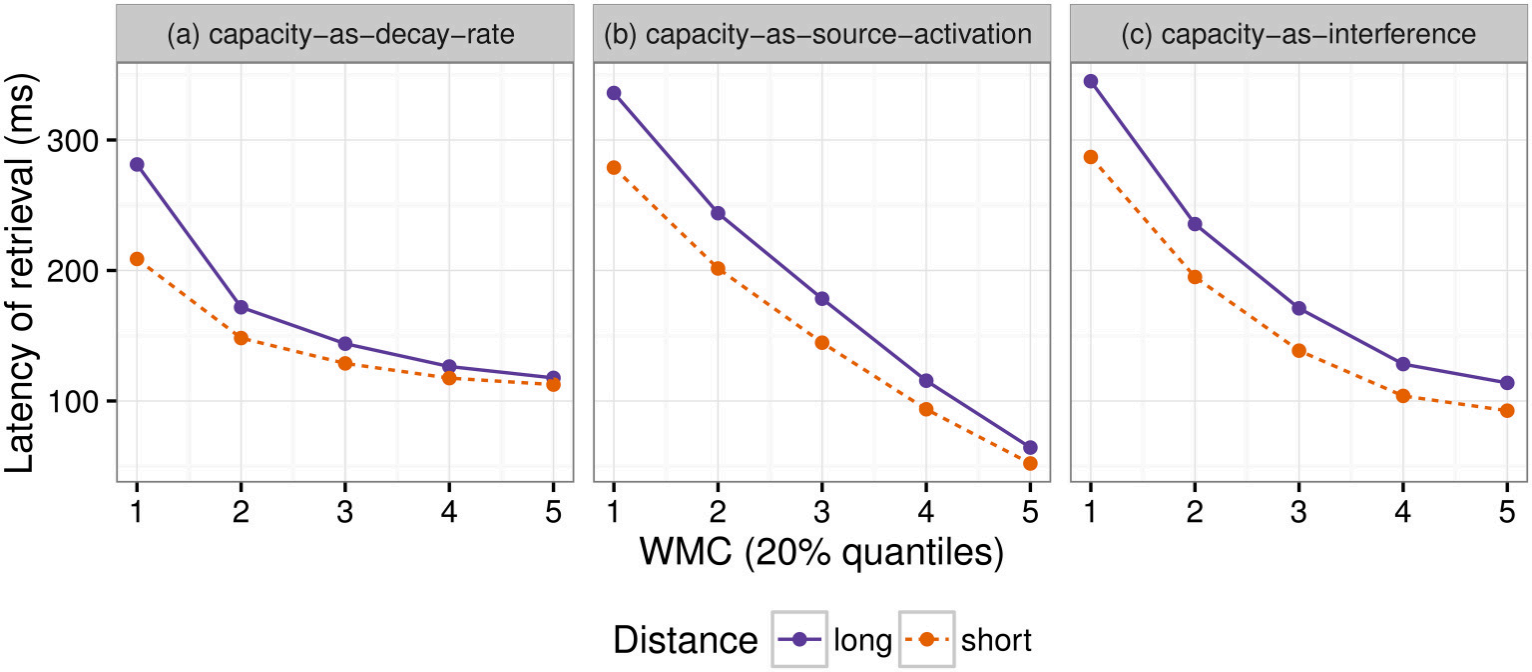
**The figures show simulated retrieval latencies at the critical region**. The simulation is based on the default but simplified version of ACT-R. See Table 6 for the parameters used.

The first possibility is the *capacity-as-decay-rate* model, which assumes that higher-WMC should predict a lower decay rate *d* (e.g., Byrne and Bovair, 1997; Cunnings and Felser, 2013). Then high-WMC participants will be less affected by longer dependency distance (which entails longer time since encoding); see Figure 7A.

If higher-WMC correlates with more spreading activation, there are two approaches: (i) The *capacity-as-source-activation* model assumes that the total amount of activation that is shared between matching cues (the source activation *W*) is a function of WMC (as in Cantor and Engle, 1993; Daily et al., 2001; van Rij et al., 2013); see Figure 7B. (ii) The *capacity-as-interference* model assumes that WMC represents susceptibility to interference (Bunting et al., 2004). Non-unique retrieval cues such as looking for a noun phrase or for the feature +*animate* cause the limited amount of source activation to be shared between competitor noun phrases, decreasing the total level of activation of the target (and also increasing the activation level of competitors). A way to model this susceptibility to interference is to change the weight given to unique cues and non-unique cues, so that as WMC increases, the weight given to a unique retrieval cue (such as being a wh-element) increases too; see Figure 7C.

These models predict mainly that an increase in WMC would increase the speed of the retrievals, as well as an interaction between WMC and dependency-head distance in raw RTs. The strength of the effect of WMC as well as the interaction will depend on the values of the parameters, and given that there is noise in the system (recall that the activation includes also a component ϵ), not every possible model will show these effects.

**Table 6:** **Parameter values for the models with the default (simplified) ACT-R**.

	**cap-as-decay-rate**	**cap-as-source activation**	**cap-as interference**
*d*	[0.06, 1.5]	0.5	0.5
*W*	1	[0.5, 3.5]	1
*w*_*wh*_	1∕3	1∕3	[0.1, 1]
*MAS*	2	2	2
*F*	1.6	1.6	1.6
σ	0.25	0.25	0.25
β	−0.65	−1	−1
(Without threshold) τ	−*Inf*	−*Inf*	−*Inf*
(With threshold) τ	0	0	0

*The decay time was calculated from the data of the German experiment; we used the mean reading time elapsed from the wh-element until the verb, which was 2614 ms for the short condition and 3922 ms for the long one*.

Given the relation between activation and latency, the models that assume that WMC affects the activation linearly (such as capacity-as-source-activation and capacity-as-interference) have two important implications: The first one is that if WMC affects the spreading activation *S*, locality effects *in raw latencies* should be modulated by WMC. The second implication is that for *log-transformed latencies*, the interaction should be exactly zero. The reason is the following: Locality effects are produced by the difference in the retrieval latencies, such that due to decay, the base level activation *BA* decreases as the distance between wh-element and head increases:

(5) $$\begin{array}{l} Locality=Latency_{LongDep}-Latency_{ShortDep}\\ \;=F\;\cdot \;(e^{-(BA_{low}+S)}-e^{-(BA_{high}+S)})\\ \;=F\;\cdot \;e^{-S}\;\cdot \;(e^{-BA_{low}}-e^{-BA_{high}}) \end{array}$$

If, as hypothesized, WMC only affects the spreading activation *S*, such that the *S* is higher for high-WMC than for low-WMC, then the interaction between locality effects and WMC would be defined as follows:

(6) $$\begin{array}{l} Locality\times WMC=Locality_{LowWMC}-Locality_{HighWMC}\\ \;=F\;\cdot \;(e^{-S_{low}}-e^{-S_{high}})\;\cdot \;(e^{-BA_{low}}-e^{-BA_{high}}) \end{array}$$

However, log-transformed locality effects are independent of *S*:

(7) $$\begin{array}{l} log(Locality)=log(F)-(BA_{low}+S)-[log(F)-(BA_{high}+S)]\\ \;=-BA_{low}+BA_{high} \end{array}$$

and thus the difference between locality effects for high and low-WMC for log-transformed latencies would be simply zero.

But critically, no matter the values of the parameters, these two models cannot predict our findings, namely, a speedup for low-span participants. This is so because the baseline activation of the wh-element when it is retrieved after a longer time (due to more intervening material between itself and the head verb) can never be higher than the level of activation when the element is retrieved after shorter time; furthermore, the spreading activation can at most attenuate this effect and only as WMC increases.

It is further assumed in ACT-R models that there is a minimum level of activation τ that an item needs in order to be retrieved. This acts as a time-out: when an item has so low activation that it would take an unrealistic amount of time to be retrieved, the retrieval fails. The maximum amount of time is a function of this activation threshold τ such that:

(8) $$\begin{array}{cc} max\left(Latency\right)=F\cdot e^{-\tau } & \; \end{array}$$

**Figure 8:**
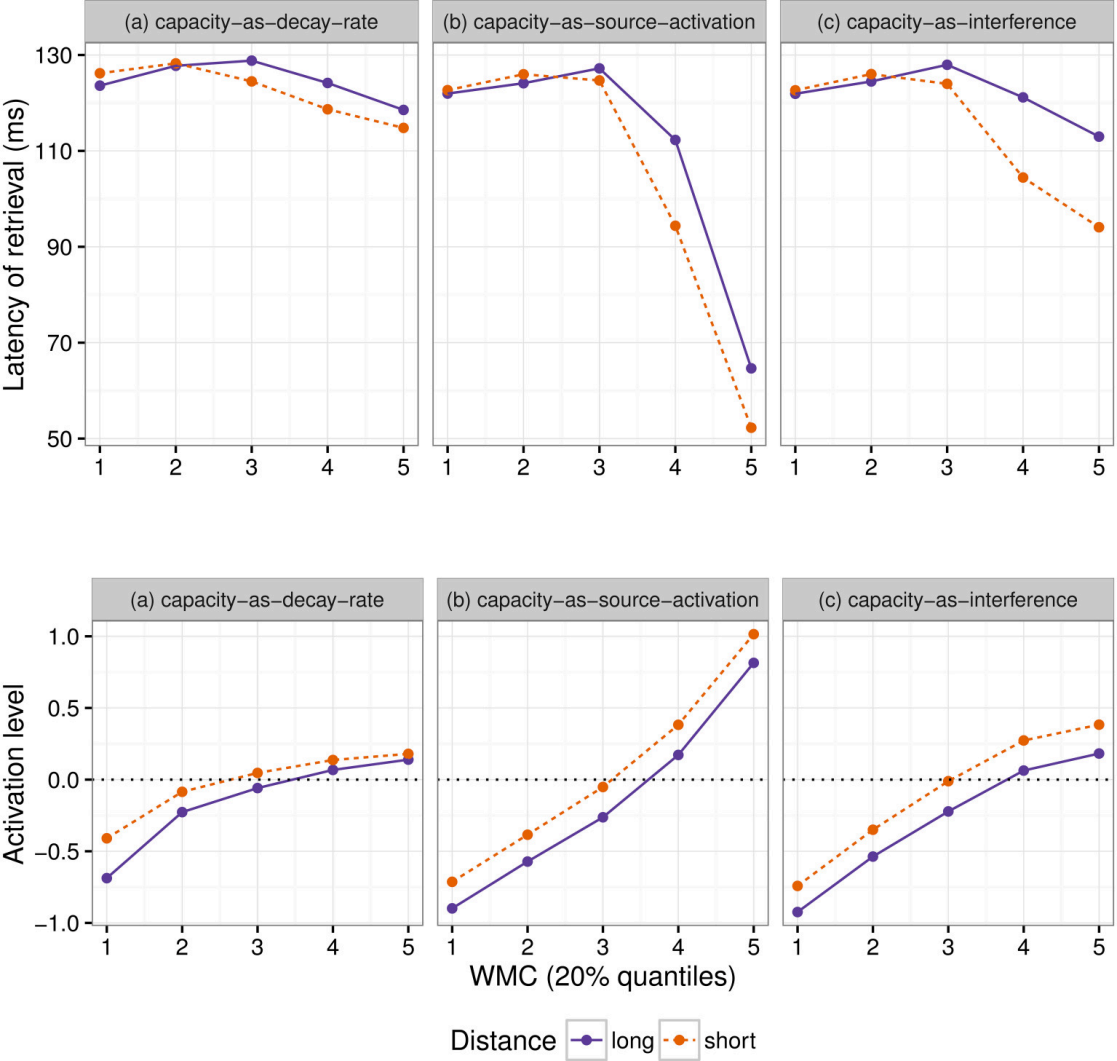
**The upper figures show simulated retrieval latencies and the lower figures the activation values that produced the latencies**. The simulation is based on the default but simplified version of ACT-R, where the threshold τ is zero. See Table 6 for the parameters used.

If τ plays a role in retrieval, because the activation level of the dependent does not always exceed this value, it will produce a ceiling effect. Under this view, if the activation level of the dependent for low-WMC failed more often than for high-WMC to surpass τ, it would entail a maximum possible time for both short and long conditions. This would produce a difference in retrieval probabilities between short and long conditions, since it is more likely that long conditions fail more often to surpass the value τ. However, this would also mean that with low-WMC, the difference between long and short conditions may disappear; see Figure 8. Our data cannot be accommodated in these models either, since the difference between long and short conditions was reversed for low-WMC.

**Figure 9:**
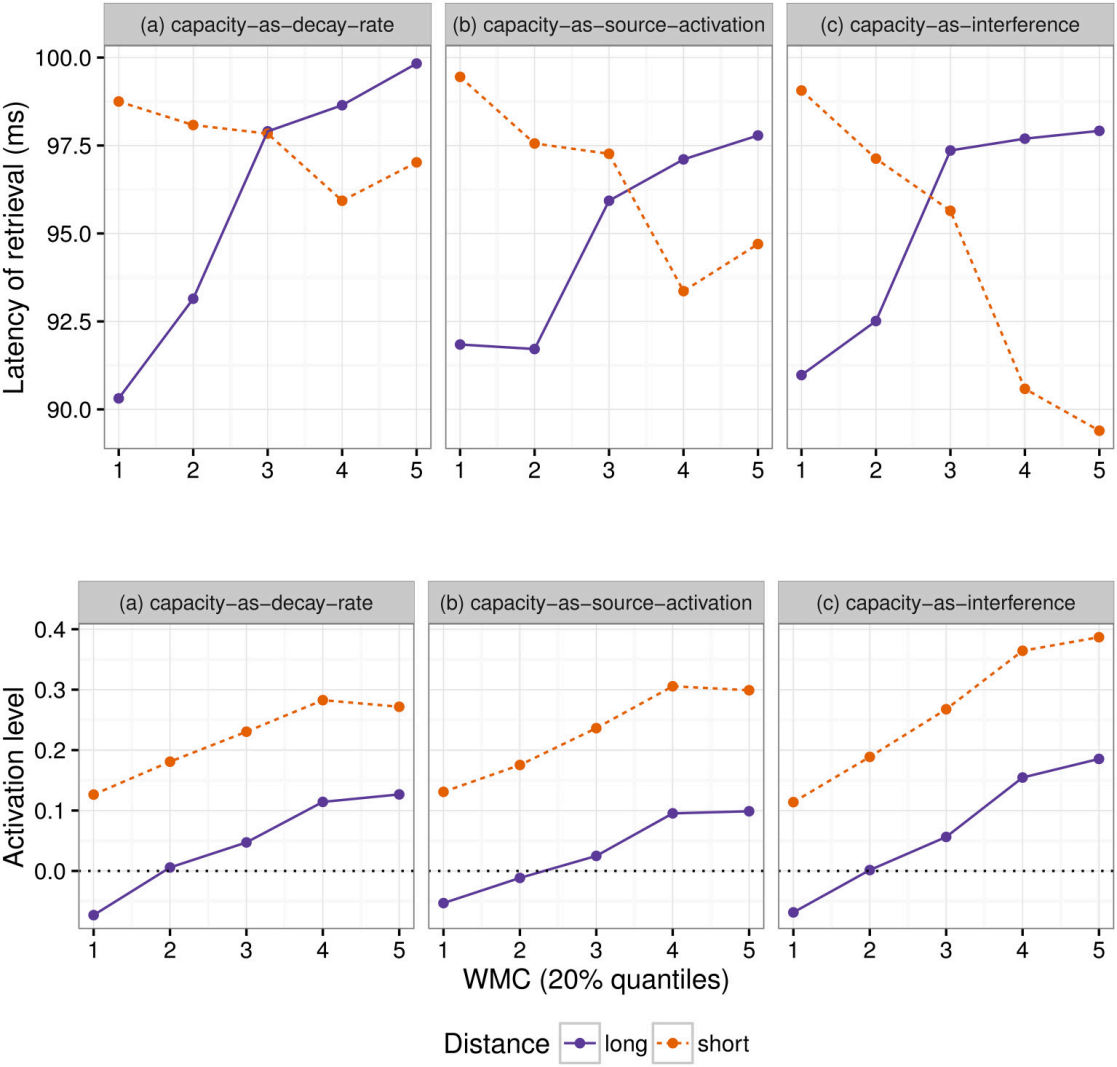
**The upper figures show simulated retrieval latencies and the lower figures the activation values that produced the latencies**. The simulation is based on the modified version of ACT-R, where the threshold τ is zero. See Table 7 for the parameters used.

The pattern that we found in our data, however, can only be accommodated in the models presented before by changing one assumption, namely, by assuming that in the cases where the activation does not reach the threshold τ, the retrieval would be aborted at any moment before the maximum amount of time. In this view, failed retrievals would take less time on average than the time needed to retrieve the item, with the activation influencing the retrieval probability and WMC in turn influencing the level of activation; see Figure 9. This would mean that τ would act as a critierion for aborting instead of a time-out. We simulated this by assuming that WMC affects the activation of the wh-element very weakly and, critically, that retrievals can fail at any time before the maximum retrieval latency (i.e., following a uniform distribution limited between zero and *max*(*Latency*)). There are, of course, other possibilities that will fit with the general pattern as well: Any distribution of latencies with a mean that is smaller than the average latency for a retrieval will show this pattern. Importantly, by relaxing the ACT-R assumption that too low activation must produce the longest possible latency, we are able to account qualitatively for the pattern in our data. This is so, because participants with lower-WMC would fail more often than high-WMC, and since they would complete retrievals relatively slowly, their failures would be on average faster. An interesting prediction from this modification is that in the small number of cases where a retrieval would fail for high-WMC participants, because high-WMC subjects should produce faster than average retrievals, they would still show slower failures in comparison to their retrievals.

There is some parallelism between fast failures in our experiment and fast errors in two-alternative forced choice tasks. Recent research in two-alternative forced choice tasks has shown that time-varying collapsing thresholds (e.g., Frazier and Yu, 2008; Drugowitsch et al., 2012; Thura et al., 2012) can explain wrong answers that are given too early, even though there is no apparent imposed deadline. Self-paced reading presents a paradigm, however, where the only possible choice at every point is to press the space bar to continue reading. In order to build a complete representation of the sentences, participants reading the verb region should delay pressing the space bar, until they retrieve from memory the dependent and they complete the dependency. However, we have argued that, when the dependent does not have enough activation, retrieval processes are aborted early. Assuming time has a cost, Frazier and Yu (2008) argue that an optimal stopping rule for a process is to stop the first time that the expected cost of continuing exceeds that of stopping, and to continue only if it is going to improve the chances of success enough to offset the extra time. A stopping rule in self-paced reading would mean pressing the space bar and continue reading. When an item to be retrieved has enough activation, an optimal stopping rule could be to wait and continue reading only when the retrieval is finished. Alternatively, when an item has insufficient activation, the parser could evaluate that the activation would not be enough to finish the retrieval before a time out (*F* · *e*^−τ^), abort the process, and continue reading, explaining the fast failures.

**Table 7:** **Parameter values for the models with the modified (simplified) ACT-R**.

	**cap-as-decay-rate**	**cap-as-source-activation**	**cap-as-interference**
*d*	[0.33, 0.62]	0.5	0.5
*W*	1	[0.9, 1.2]	1
*w*_*wh*_	1∕3	1∕3	[0.2, 0.5]
*MAS*	2	2	2
*F*	1.6	1.6	1.6
σ	0.25	0.25	0.25
β	−0.3	−0.3	−0.3
(With threshold) τ	0	0	0

*The decay time was calculated from the data of the German experiment; we used the mean reading time elapsed from the wh-element until the verb, which was 2614 ms for the short condition and 3922 ms for the long one*.

Further research with data that include RTs as well as some index of retrieval accuracy, which is as little contaminated as possible with general comprehension accuracy, other retrievals, and offline processes, could shed light on how and when exactly retrieval fails.

## 6. Conclusion

We presented two experiments showing that working memory affects locality effects. The results show that working memory affects retrieval times at unbounded dependency resolution, but in an unexpected manner: high-capacity readers showed the strongest locality effects that decreased with decreasing capacity and eventually changed direction, such that low-capacity readers showed antilocality effects.

We suggest that the results may not be simply due to a speed-accuracy trade-off and that they can be explained by adding two assumptions to memory-based explanations: (i) compared to high-capacity readers, low-capacity readers experience retrieval failures more frequently; and (ii) retrieval failures are on average faster than complete retrievals. We suggest that the retrieval failures end quickly because of insufficient activation, and this activation depends not only on dependent-head distance but also on the capacity of the readers.

All in all, both experiments show that translating longer RTs into processing difficulty and shorter RTs into facilitation may be too simplistic, especially when readers face long and complex sentences (which are not uncommon in psycholinguistic studies). Our results suggest that the same increase in processing difficulty may lead to slowdowns in high-capacity readers and speedups in low-capacity ones.

## Funding

The work was supported by Minerva Foundation, Potsdam Graduate School, and the University of Potsdam. We acknowledge the support of the Deutsche Forschungsgemeinschaft and Open Access Publishing Fund of University of Potsdam.

### Conflict of interest statement

The authors declare that the research was conducted in the absence of any commercial or financial relationships that could be construed as a potential conflict of interest.

## Supplementary material

Data and complete code are available in the website of the first author.
