# Supplementary material for: When High-Capacity Readers Slow Down and Low-Capacity Readers Speed Up: Working Memory and Locality Effects
Source: Front Psychol. 2016 Mar 8;7:280. doi: 10.3389/fpsyg.2016.00280 (PMC4782223; doi:10.3389/fpsyg.2016.00280)
Supplement: Supplementary file 1 [file Table1.PDF]

Frontiers in Psychology

Research Article
December 26, 2015

Supplementary Material: When high-capacity readers slow down and low-capacity readers speed up: Working memory and locality effects

Nicenboim, Bruno [1,*], Logačev, Pavel [2], Gattei, Carolina,[3] and Vasishth, Shravan [4]

[1] *Department of Linguistics, University of Potsdam, Potsdam, Germany*
[2] *Department of Linguistics, University of Potsdam, Potsdam, Germany*
[3] *Grupo de Lingstica y Neurobiologa Experimental del Lenguaje, INCIHUSA, CONICET, Mendoza, Argentina.*
[4] *Department of Linguistics, University of Potsdam, Potsdam, Germany*

Correspondence*:
Bruno Nicenboim
Department of Linguistics, University of Potsdam, Karl-Liebknecht-Str. 24-25, D-14476 Potsdam, Germany, bruno.nicenboim@uni-potsdam.de

Encoding and navigating linguistic representations in memory

1 SUPPLEMENTARY TABLES

For comparison purposes, we provide the results of the frequentist linear mixed-effects models (LMM; **Pinheiro and Bates**, 2000) using the *lme4* package (**Bates et al.**, 2014) in Tables 1 and 2 . The models were fit with maximal random effects structure justified by the design removing the correlation between variance components to ensure convergence. We transformed the dependent variable (RT) using the reciprocal transformation (Box-Cox method: **Box and Cox**, 1964), but we used $(-10^5/RT)$ to improve the readability of the models (a positive t-value for $-10^5/RT$ corresponds to a positive t-value of the untransformed measure RT). Extreme values of RTs were removed from the analysis (either below 150ms or above 5000ms).

Table 1. LMMs for Spanish experiment

Predictor	Estimate	*SE*	*t*-value
length	0.02	0.01	1.87
dependency	-0.01	0.01	-0.87
WMC	0.00	0.07	0.01
RF	-0.28	0.07	-4.05
length:dependency	0.01	0.01	0.67
length:dependency:WMC	0.03	0.01	2.76
length:dependency:RF	-0.01	0.01	-1.28

Table 2. LMMs for German experiment

Predictor	Estimate	*SE*	*t*-value
length	-0.01	0.01	-1.33
dependency	-0.01	0.01	-1.47
WMC	0.02	0.07	0.27
RF	-0.15	0.07	-2.26
length:dependency	0.01	0.01	0.72
length:dependency:WMC	0.02	0.01	1.95
length:dependency:RF	-0.01	0.01	-1.32

2 STAN CODE

We used the following code to fit the shifted lognormal mixed models for the analysis of RTs.

```

functions {
  real shift_max(vector shift_u, int[] subj, vector rt) {

    real shift_max;
    shift_max <- positive_infinity();
    for (i in 1:num_elements(rt)){
         shift_max <- fmin(shift_max,log(rt[i]) - shift_u[subj[i]] );
        }
    return shift_max;
  }

  real shifted_lognormal_log(vector y, vector mu, real sigma, vector psi){

    if (min(psi) < 0)
      reject("Shift parameter (psi) should be bigger than 0, value found ",
        min(psi));

    if (min(y-psi) < 0)
      reject(
"Shift parameter (psi) should be smaller than y;  y-psi > 0 but here there is",
min(y-psi));

  return lognormal_log(y- psi,mu,sigma);
  }

}


data {
  int<lower=0> N_obs;
  int<lower=0> N_coef;
  int<lower=0> N_coef_u;
  int<lower=0> N_coef_w;
  int<lower=0> N_coef_v;
  int<lower=1> subj[N_obs];    //subject id
  int<lower=1> item[N_obs];    //item id
  int<lower=1> sentence[N_obs];    //sentence id

  int<lower=1> N_subj;
  int<lower=1> N_item;
  int<lower=1> N_sentence;
  matrix[N_obs,N_coef] x;
```

```
  matrix[N_obs,N_coef_u] x_u;
  matrix[N_obs,N_coef_w] x_w;
  matrix[N_obs,N_coef_v] x_v;
  vector[N_obs] rt;

}

transformed data {
  matrix[N_obs,N_coef-1] x_betas;
  x_betas <- block(x,1,2,N_obs,N_coef-1); # I remove the intercept here
}

parameters {
  vector[N_coef-1] delta;     //delta is size of the effect
  real<lower=0> sigma;
  real<lower=0> tau_shift;
  vector[N_subj] shift_u_raw;  // subj shift
  real<upper=shift_max(shift_u_raw * tau_shift,subj,rt)> shift;

  //subj
  vector<lower=0> [N_coef_u]  tau_u;     // subj sd
  cholesky_factor_corr[N_coef_u] L_u;      // correlation matrix for random intercepts and slopes subj
  vector[N_coef_u] z_u[N_subj];

  //items
  vector<lower=0> [N_coef_w]  tau_w;     // subj sd
  cholesky_factor_corr[N_coef_w] L_w;      // correlation matrix for random intercepts and slopes item
  vector[N_coef_w] z_w[N_item];

  //sentence
  vector<lower=0> [N_coef_v]  tau_v;     // subj sd
  cholesky_factor_corr[N_coef_v] L_v;      // correlation matrix for random intercepts and slopes item
  vector[N_coef_v] z_v[N_sentence];

  real<lower=0> alpha;

}

transformed parameters {

  vector[N_coef_u]  u[N_subj];
  vector[N_coef_w]  w[N_item];
  vector[N_coef_v]  v[N_sentence];
  matrix[N_coef_u,N_coef_u] Lambda_u;
  matrix[N_coef_w,N_coef_w] Lambda_w;
  matrix[N_coef_v,N_coef_v] Lambda_v;
  vector[N_coef-1] beta;
  vector[N_obs] psi;  //each shift
  vector[N_obs] mu;
  vector[N_subj] shift_u;  // subj shift

  beta <- delta * sigma;

  Lambda_u <- diag_pre_multiply(tau_u,L_u);
  for (i in 1:N_subj){
   u[i] <- Lambda_u * z_u[i];
  }

  Lambda_w <-  diag_pre_multiply(tau_w,L_w);
  for (i in 1:N_item){
    w[i] <- Lambda_w * z_w[i]; // item random effects
  }

```

```
  Lambda_v <-  diag_pre_multiply(tau_v,L_v);
  for (i in 1:N_sentence){
    v[i] <- Lambda_v * z_v[i]; // item random effects
  }


  shift_u <- shift_u_raw * tau_shift; // =shift_u ~normal(0,tau_shift)
  for (i in 1:N_obs){
    mu[i] <- alpha + x_betas[i] * beta +
          x_u[i] * u[subj[i]] +
          x_w[i] * w[item[i]] +
          x_v[i] * v[sentence[i]];
    psi[i] <- exp(shift + shift_u[subj[i]]); //
  }

}


model {

  sigma ~ normal(0,1);
  tau_u ~ normal(0,1);
  tau_w ~ normal(0,1);
  tau_v ~ normal(0,1);
  tau_shift  ~ normal(0,.5);

  alpha ~ normal(0, 5);
  delta ~ normal(0, .2);

  #for (i in 1:N_subj)
  shift ~ normal(0,1);
  shift_u_raw ~ normal(0,1);

  L_u ~ lkj_corr_cholesky(4.0);
  L_w ~ lkj_corr_cholesky(4.0);
  L_v ~ lkj_corr_cholesky(4.0);

  for (i in 1:N_subj){
    z_u[i] ~ normal(0,1);
  }

  for (i in 1:N_item){
    z_w[i] ~ normal(0,1);
  }

  for (i in 1:N_sentence){
    z_v[i] ~ normal(0,1);
  }

    rt ~ shifted_lognormal(mu, sigma,psi);
}

```

We used the following code to simulate the default implementation of ACT-R.

```
data {
  int<lower=0> N_obs;
  real<lower=0> sigma;
  real expF;
  real<lower=0> W_slope;
  real weight_slope;
```

```
  vector[N_obs] wmc;
  real<lower=> W_int;
  real<lower=log(5)> MAS;
  real beta;
  real tau_int;  #
  vector[N_obs] decay_time;
  real<upper=1> d;
  real d_slope;

}

transformed data {
 real shared_cues;
 shared_cues <- 5.0;
}

model {
}

generated quantities {
  vector[N_obs]  Base_level_activation;
  vector<lower=0>[N_obs]  Spreading_activation;
  vector[N_obs]  A;
  vector<lower=0> [N_obs]  W;
  vector[N_obs]  tau;
  real s;
  vector<lower=0>[N_obs]  Pr;
  vector<lower=0,upper=1>[N_obs] weight;
  vector<upper=0>[N_obs] d;

  real pred_latency[N_obs];
  real pred_accuracy[N_obs];


  for (i in 1:N_obs){
    d[i] <- -exp(log(d)+d_slope* wmc[i]);
    Base_level_activation[i] <- log(decay_time[i]^d[i] ) +beta;
    W[i] <- exp(log(W_int) + W_slope * wmc[i]);
    tau[i] <- tau_int;
    weight[i] <- inv_logit(logit(1.0/3) + weight_slope * wmc[i]);
    Spreading_activation[i] <- W[i] * (weight[i] * (MAS-log(1)) + //unique cue
                        (1-weight[i]) * (MAS-log(shared_cues))) ;//non-unique cues
  }

  A <- Base_level_activation+Spreading_activation;
  s <- sqrt(3.0)*sigma/pi();
  Pr <- 1.0 ./(1.0+exp(-(A-tau)/s) );

  for (i in 1:N_obs){
    pred_accuracy[i] <- bernoulli_rng(Pr[i]);
    if(pred_accuracy[i]==1) {
      pred_latency[i] <- exp(logistic_rng(-A[i] + expF, sigma));
    } else {
      pred_latency[i] <- exp(-tau[i]+ expF)  ;
    }
  } //for
} // generated quantities
```

In our modified implementation of ACT-R only the predicted latency is changed:

```
for (i in 1:N_obs){
pred_accuracy[i] <- bernoulli_rng(Pr[i]);
  if(pred_accuracy[i]==1) {
    pred_latency[i] <- exp(logistic_rng( -A[i] + expF , sigma));
  } else {
    pred_latency[i] <- uniform_rng(0,exp(-tau[i]+ expF))  ;
  }
} //for
```

REFERENCES

Bates, D. M., Mächler, M., Bolker, B. M., and Walker, S. C. (2014), Fitting Linear Mixed-Effects Models using lme4, *Journal of Statistical Software (forthcoming)*

Box, G. E. P. and Cox, D. R. (1964), An analysis of transformations, *Journal of the Royal Statistical Society. Series B (Methodological)*, 211–252

Pinheiro, J. C. and Bates, D. M. (2000), Linear Mixed-Effects Models: Basic Concepts and Examples, in Mixed-Effects Models in S and S-PLUS (Springer New York), Statistics and Computing, 3–56
